# Reconstructing Kinetic Models for Dynamical Studies of Metabolism using Generative Adversarial Networks

**DOI:** 10.1038/s42256-022-00519-y

**Published:** 2022-08-30

**Authors:** Subham Choudhury, Michael Moret, Pierre Salvy, Daniel Weilandt, Vassily Hatzimanikatis, Ljubisa Miskovic

**Affiliations:** 1 Laboratory of Computational Systems Biology (LCSB), Ecole Polytechnique Fédérale de Lausanne (EPFL), Lausanne, Switzerland; 2 Present Address: Cambrium GmBH, Berlin, Germany; 3 Present Address: Lewis-Sigler Institute for Integrative Genomics, Princeton University, Princeton, NJ USA

**Keywords:** Nonlinear dynamics, Computational models, Metabolic engineering, Dynamical systems

## Abstract

Kinetic models of metabolism relate metabolic fluxes, metabolite concentrations and enzyme levels through mechanistic relations, rendering them essential for understanding, predicting and optimizing the behaviour of living organisms. However, due to the lack of kinetic data, traditional kinetic modelling often yields only a few or no kinetic models with desirable dynamical properties, making the analysis unreliable and computationally inefficient. We present REKINDLE (Reconstruction of Kinetic Models using Deep Learning), a deep-learning-based framework for efficiently generating kinetic models with dynamic properties matching the ones observed in cells. We showcase REKINDLE’s capabilities to navigate through the physiological states of metabolism using small numbers of data with significantly lower computational requirements. The results show that data-driven neural networks assimilate implicit kinetic knowledge and structure of metabolic networks and generate kinetic models with tailored properties and statistical diversity. We anticipate that our framework will advance our understanding of metabolism and accelerate future research in biotechnology and health.

## Abstract (web summary)

Kinetic models of metabolism capture time-dependent behaviour of cellular states and provide valuable insights into cellular physiology, but, due to the lack of experimental data, traditional kinetic modelling can be unreliable and computationally inefficient. A generative framework based on deep learning called REKINDLE can efficiently parameterize large-scale kinetic models, enabling new opportunities to study cellular metabolic behaviour.

## Main

Technological progress in high-throughput measurement techniques has propelled discoveries in biotechnology and medicine and allowed us to integrate different data types into representations of cellular states and obtain insights into cellular physiology. Historically, researchers have used genome-scale models (mathematical descriptions of cellular metabolism) to associate experimentally observed data with cellular phenotype^1–3^. However, traditional genome-scale models cannot predict the dynamic cellular responses to internal or external stimuli because they lack information about metabolic regulation and enzyme kinetics^4,5^. Recently, the research community has shifted focus to developing kinetic metabolic models to advance our understanding of cellular physiology^5^.

Kinetic models capture time-dependent behaviour of cellular states, providing additional information about cellular metabolism compared with that obtained with steady-state methods such as flux balance analysis^6–8^. However, the difficulty of acquiring the knowledge of (1) the exact mechanism of each reaction and (2) the parameters of the said mechanisms, such as Michaelis constants or maximal velocities, hampers the building of kinetic models. In most kinetic modelling methods^9–12^, the unknown reaction mechanisms are hypothesized or modelled by approximate reaction mechanisms^13,14^. The main challenge in obtaining the unknown parameters is uncertainties intrinsic to biological systems. Due to the inherently underdetermined nature of the mathematical equations describing the biological systems, the model can often reproduce the experimental measurements for multiple rather than a unique set of parameter values. To address these challenges, the researchers frequently employ frameworks based on Monte Carlo sampling^15–19^. In these approaches, we first reduce the space of admissible parameter values by integrating the experimental measurements and ensuring consistency with the physicochemical laws. The reduced solution space is then sampled to extract alternative parameter sets.

**Fig. 1:**
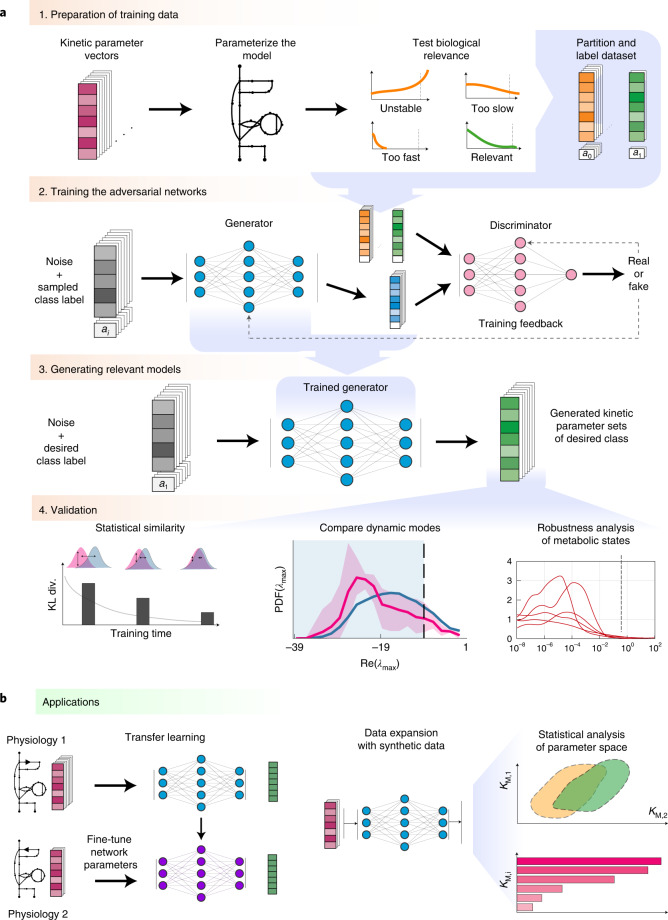
Overview of the REKINDLE framework and applications. **a**, Framework. Step 1—kinetic parameter sets are tested for prespecified conditions (models that describe experimentally observed data and have appropriate dynamic properties) and are labelled and partitioned into data classes. Step 2—REKINDLE employs GANs to learn the distribution of labelled data obtained from the previous step. Step 3—a trained generator from the GAN generates new kinetic parameters of models that satisfy prespecified conditions. Step 4—the generated dataset is subjected to statistical and validation tests to determine the fulfilment of the imposed conditions. **b**, Applications. Left: REKINDLE uses the specifics of physiological and structural knowledge acquired by the GANs during training to extrapolate to other physiologies via transfer learning when training data is limited. Right: the REKINDLE-generated kinetic parameter sets are amenable to extensive and advanced statistical analysis, allowing further insights into studied phenotypes to be revealed. *K*_m,1_ and *K*_m,2_ represent any two kinetic parameters.

However, sampling-based kinetic modelling frameworks frequently produce large subpopulations of kinetic models inconsistent with the experimentally observed physiology. For instance, the constructed models can be locally unstable or display too fast or too slow time evolution of metabolic states compared with the experimental data (Fig. 1). This entails a considerable loss of computational efficiency, especially for the low incidence of subpopulations with desirable properties. For example, the generation rate of locally stable large-scale kinetic models can be lower than 1% (ref. ^20^). Requiring other model properties such as experimentally observed time evolution of metabolic states further reduces the incidence of desired models. Indeed, just a tiny fraction of the parameter space satisfies all desirable model properties simultaneously, and our observations suggest that this subspace is not contiguous. Moreover, none of these methods guarantees that the sampling process, often implemented as unbiased samplifng, will produce the desirable parameter sets. These drawbacks become amplified with increasing size of the kinetic models, and finding regions in the parameter space that satisfy the desired properties and observed physiology becomes challenging. Additionally, the structure of these regions is so complex that nonlinear function approximators such as neural networks are required to map them (Supplementary Notes 1 and 2).

We present REKINDLE (Reconstruction of Kinetic Models using Deep Learning) to address these challenges. This unsupervised deep-learning-based method leverages generative adversarial networks (GANs)^21^ to generate kinetic models that capture experimentally observed metabolic responses. REKINDLE utilizes existing kinetic modelling frameworks to create the data required for the training of GANs. Efficient generation of models with desired properties using these neural networks (Fig. 1a) substantially reduces the need for the extensive computational resources required by the traditional kinetic modelling methods. For example, REKINDLE can be used to create large synthetic datasets within a matter of seconds on commonly used hardware. Importantly, we showcase REKINDLE’s ability to navigate through the physiological states of metabolism using transfer learning^22,23^ in the low-data regime, demonstrating that the neural networks trained for one physiology can be fine-tuned for another physiology using a small amount of data (Fig. 1b). REKINDLE’s departure from the traditional way of creating kinetic models paves the way for more comprehensive computational studies and advanced statistical analysis of metabolism.

## REKINDLE for generation of biologically relevant kinetic models

The REKINDLE framework consists of four successive steps (Fig. 1a). The inputs of REKINDLE are kinetic parameter sets obtained from traditional kinetic modelling methods—for example, via Monte Carlo sampling^9,10,17,18,24^. The procedure starts by testing the biological relevance of the kinetic parameter sets. We consider that a kinetic parameter set is biologically relevant if the metabolic responses obtained from the kinetic model with this parameter set have experimentally observed dynamic responses (Methods). We then categorize the parameter sets into two classes, biologically relevant or not relevant (for example, sets providing metabolic responses with too slow, too fast or unstable dynamics), and label them accordingly (Fig. 1a, step 1). While here we use REKINDLE to generate kinetic models with biologically relevant dynamics, the framework allows imposition of other biochemical properties or combinations of properties and physiological conditions to construct and label the dataset. A labelled dataset is then used to train the conditional GANs^25^ (Fig. 1a, step 2).

Conditional GANs consist of two feedforward neural networks, the generator and the discriminator, which are conditioned on class labels during training. The goal of the training procedure is to obtain a good generator that generates kinetic models (Fig. 1a, step 3) from a specific predefined class that are indistinguishable from the kinetic models of the same class in the training data (Methods).

Once the training is done, we validate the biological relevance of the generated kinetic models via a series of tests (Fig. 1a, step 4). We first test the statistical similarity of the generated and training data by comparing their distributions in the parameter space. We then check the distributions of the eigenvalues of the Jacobian (Methods) and their corresponding dominant time constants to verify if the generated parameter sets satisfy the desired dynamic responses. Finally, we test the models’ dynamic responses to perturbations in the steady-state metabolic profile to evaluate the robustness of the generated parameter sets.

## Results

### REKINDLE generates kinetic models of *Escherichiacoli* metabolism

We showcase REKINDLE by generating biologically relevant kinetic models of the *E. coli* central carbon metabolism (Supplementary Fig. 1). The models are parameterized with 411 kinetic parameters (Methods). Thermodynamic-based flux analysis^26–28^ performed on the model with the integrated experimental data from aerobic cultivation of wild-type *E. coli*^29^ indicated that two reactions, transaldolase (TALA) and isocitrate lyase (ICL), could operate in both forward and reverse directions, whereas the other reactions had unique directionalities (Table 1 and Methods). This means that the study of this physiological condition requires generation of four populations of kinetic models, with each population corresponding to a different combination of TALA and ICL directionalities. We enumerated these four cases as physiologies 1–4 (Table 1).

**Table 1:** Incidence of biologically relevant models generated with ORACLE (training data) and REKINDLE for four physiologies

	Physiology 1	Physiology 2	Physiology 3	Physiology 4
Directionality of reactions	$\underrightarrow {\mathrm{TALA}}$ $\underrightarrow {\mathrm{ICL}}$	$\underrightarrow {\mathrm{TALA}}$ $\underleftarrow {\mathrm{ICL}}$	$\underleftarrow {\mathrm{TALA}}$ $\underrightarrow {\mathrm{ICL}}$	$\underleftarrow {\mathrm{TALA}}$ $\underleftarrow {\mathrm{ICL}}$
ORACLE (training data)	58.8%	55.0%	61.1%	56.0%
REKINDLE	97.7%	97.3%	99.3%	100%

The physiologies differ in the directions in which TALA and ICL operate. TALA transforms glyceraldehyde-3-phosphate to d-fructose 6-phosphate in the pentose phosphate pathway, and ICL converts isocitrate to succinate and glyoxylate in the tricarboxylic acid cycle. The REKINDLE results represent the maximal incidence achieved for five repeats.

While REKINDLE can use parameter sets of any kinetic modelling framework for the training, we employed ORACLE^20,24,30–34^ implemented in the SKiMpy toolbox^35^ to generate a training dataset of 72,000 parameter sets for each physiology. The goal was to generate kinetic models that satisfy the observed steady state and have dynamic responses that are faster than 6–7 min (which corresponds to one third of the *E. coli* doubling time^36^) (Methods). The kinetic models satisfying these conditions can reliably reproduce experimentally measured metabolic responses in *E. coli*.

**Fig. 2:**
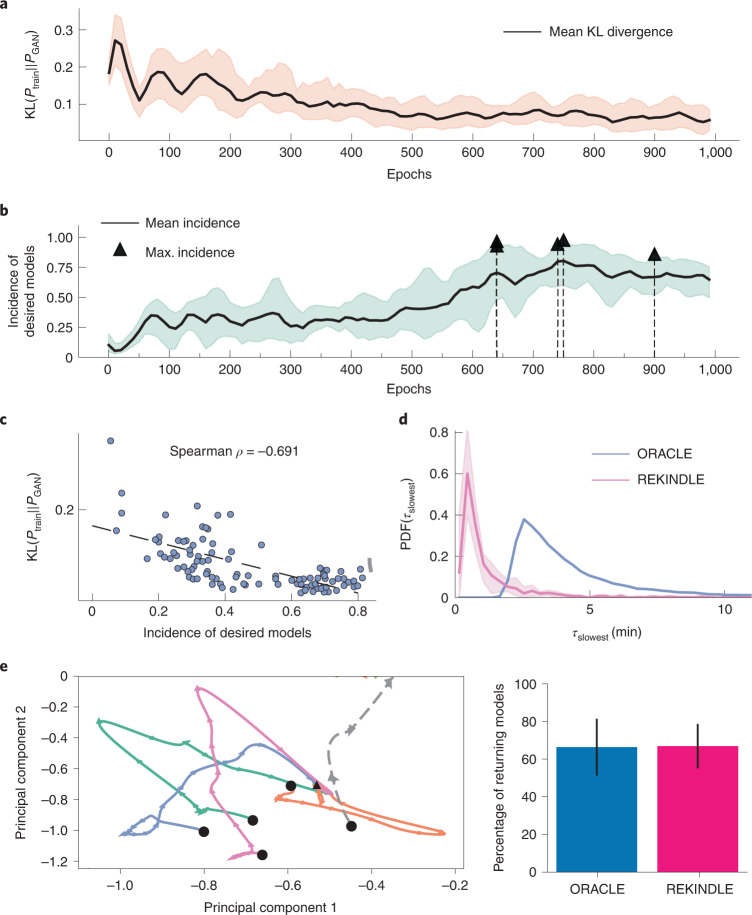
Generation and validation of GAN-generated kinetic models. **a**, The similarity between the training and generated data increases with number of training epochs, as indicated by the decreasing KL divergence between their distributions (black line) for five statistical replicates. The orange area indicates the KL divergence scores observed in five repeats. **b**, The mean incidence of biologically relevant models in the generated data during training (black line). The black triangles indicate the maximum incidence attained for each repeat (Table 1). The green area indicates the incidence of biologically relevant models observed in five repeats. **c**, Negative correlation of the mean incidence of relevant models with the KL divergence between the training and REKINDLE-generated data. **d**, REKINDLE shifts the dynamic responses of the generated data towards higher values, compared with the training data. The pink shaded area indicates overlap of distributions of *τ*_slowest_ in five repeats for the generators with the highest incidence of biologically relevant models. **e**, Perturbation analysis. Left: time evolution of the randomly perturbed metabolic levels up to ±50% represented in the PCA space for five REKINDLE-generated models. The black circles represent the initial perturbed state, the arrows indicate the directionality of the trajectory in the PCA space and the black triangle indicates the final, reference, state. The perturbed states either return to the reference steady state (orange, blue, magenta, green) or escape into a pathological state (grey dashed). Right: fraction of the perturbed models that return to the reference steady state for the training data produced by ORACLE- and REKINDLE-generated data.

Inspection of the training data shows that between 39% and 45% of models for the four physiologies have dynamics that are too slow (Table 1), meaning that these models cannot describe the *E. coli* metabolism. We employed REKINDLE to improve the incidence of kinetic models consistent with the *E. coli* dynamics. To this end, we trained conditional GANs for 1,000 epochs with five statistical replicates for the four physiologies. Every 10 epochs, the generator was used to generate 300 biologically relevant models. We quantified the similarity of the REKINDLE-generated and the ORACLE-generated parameters (only the parameter sets corresponding to the biologically relevant dynamics) by calculating the Kullback–Leibler (KL) divergence between the distributions of the REKINDLE-generated and training (Fig. 2a) as well as the REKINDLE-generated and test (Methods and Supplementary Note 4) datasets. Here, we present the results for physiology 1 (Table 1 and Fig. 2), whereas the results for physiologies 2–4 can be found in Supplementary Figs. 3a–c and 4a–c.

The KL divergence decreased with training, meaning that the GAN learns the distribution of the kinetic parameters that correspond to biologically relevant dynamics (Fig. 2a and Supplementary Notes 4 and 8). The decrease in the KL divergence score also indicated that the GAN is not suffering from mode collapse, a common pathology in GANs, where the generator maps the entire latent space to a small region in the feature space^37^. Additionally, the GAN was not subject to overfitting (Supplementary Note 3). We also tested the generated models for biological relevance using linear stability analysis of the resulting parameterized system of ordinary differential equations (ODEs) (Methods). The incidence of relevant models increased with the number of training epochs, reaching as high as 0.977 (97.7% of the generated models) for some repeats (Fig. 2b and Table 1). Moreover, a negative correlation of *ρ* = −0.691 (Spearman correlation coefficient) between the number of relevant models at a given epoch and the KL divergence (Fig. 2c) indicated that the KL divergence is a good measure for assessing the training quality.

The training stabilized after ~400 epochs with the discriminator accuracy around 50% (Supplementary Fig. 2a–d), suggesting that the generated models were not an artefact of a failed training process (Methods). The peak incidence of desired models occurred at different numbers of epochs for different replicates (Fig. 1b).

### Validation of the REKINDLE-generated models

We next performed additional validation checks to determine the quality of the generated kinetic models. For all checks, we chose the generator with the highest incidence of desired models (Fig. 2b) and used it to generate 10,000 biologically relevant kinetic models.

We first verified how fast were the dynamics of generated models by computing the distribution of the dominant characteristic time constant of the dynamic responses, *τ*, resulting from the generated kinetic parameter sets (Methods). The distribution of the time constants of the REKINDLE-generated models has shifted towards smaller values compared with the distribution of the time constants of the models from the training set (Fig. 2d). This meant that REKINDLE-generated models have faster dynamical responses than do those from the training set (Supplementary Note 5). Indeed, most of the REKINDLE-generated models have a dominant time constant of ~1 min, indicating that the metabolic processes settle before the subsequent cell division (doubling time of ~21 min, ref. ^36^). In contrast, most of the models from the training set have a dominant time constant of 2.5 min, with the distribution having a heavy tail towards longer time constants (Fig. 2d). We have observed similar results for the three remaining physiologies (Supplementary Fig. 3a–c).

We next compared the robustness of the REKINDLE-generated and ORACLE-generated kinetic models by perturbing the steady state and verifying if the perturbed system would evolve back to the steady state (Fig. 2e). For this purpose, we randomly chose 1,000 biologically relevant parameter sets from training and generated datasets, and we perturbed the reference steady state, *X*_RSS_, with random perturbations, Δ*X*, between 50% and 200% of the reference steady state (*X*_RSS_/2 ≤ Δ*X* ≤ 2*X*_RSS_). We repeated this procedure 100 times for each of the 1,000 models. In most cases, the system returns to within 1% of the steady state at the *E. coli* doubling time, indicating that the kinetic models are locally stable around the reference steady state and satisfy the imposed dynamic constraints. Indeed, the fractions of models returning to the steady state are comparable for REKINDLE (66.85%) and ORACLE (66.31%) (Fig. 2e, right). For the remaining three physiologies, REKINDLE-generated models were consistently more robust than those generated by ORACLE. For example, for physiology 4, 83.79% of the REKINDLE-generated models return to the steady state, compared with 61.05% of the ORACLE-generated models (Supplementary Fig. 4a–c).

To visualize the time evolution of the perturbed state of the kinetic models, we performed principal component analysis (PCA) on the time-series data of the ODE solutions. The first two principal components explained 97.17% of the total variance in the solutions (component 1, 85.21%; component 2, 11.95%). We plotted these components for four randomly selected REKINDLE-generated kinetic models that returned to the reference steady state and one that escaped (Fig. 2e, left).

Thus, REKINDLE reliably generates kinetic models robust to perturbations and obeying biologically relevant time constraints.

### Interpretability of the REKINDLE-generated models

**Fig. 3:**
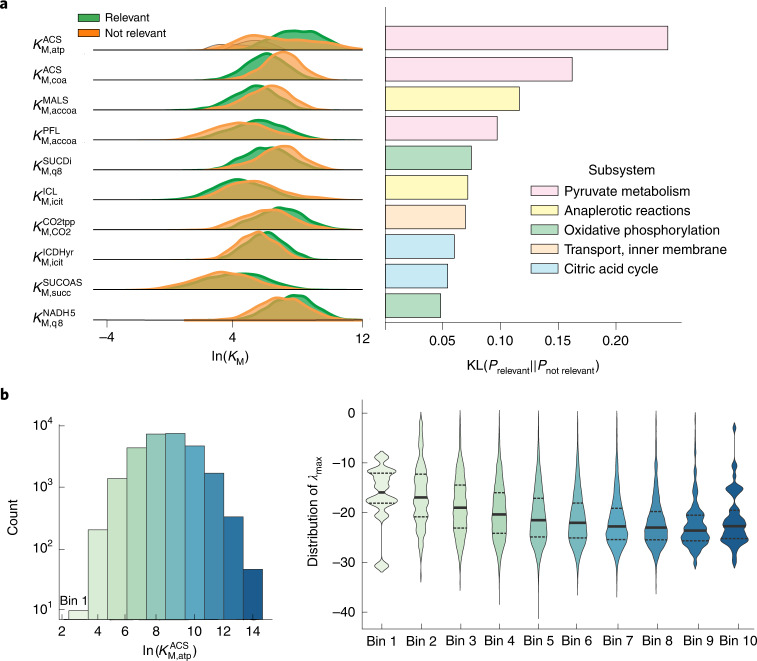
Interpretability of REKINDLE-generated sets. **a**, Left: probability distribution comparison between relevant and not relevant kinetic models for the top ten ranked kinetic parameters (Supplementary Fig. 6 and Supplementary Table 1). The parameters are ranked by the highest KL divergence between the two distributions. Right: respective magnitudes of KL divergence for the ten parameters shown in **a**. **b**, Left: 50,000 REKINDLE-generated models were divided into ten subpopulations (indicated by the different shades of the bins) on the basis of their respective values of $K_{{\mathrm{M}},{\mathrm{atp}}}^{\mathrm{ACS}}$. Right: maximum eigenvalue distributions of the Jacobian for the subpopulations. The thick black line indicates the mean of the distribution and the dashed lines indicate quartiles.

We used KL divergence to compare the distributions of biologically relevant and irrelevant kinetic models (2,000 kinetic models from each category) for physiology 1 and identify the kinetic parameters that affect biological relevance. Only a handful of parameters substantially differed in the distributions between the two populations (Supplementary Fig. 5a,b), which is consistent with studies showing that only a few kinetic parameters affect specific model properties^38,39^, whereas most parameters are sloppy^40^. We inspected distributions of the top ten parameters with the highest KL divergence score (Fig. 3a). There was a clear bias in the distributions of the two populations, suggesting that these parameters are indeed affecting the biological relevance of the generated models (Supplementary Note 6).

On the basis of these results, we hypothesized that the values of the top parameter, $K_{{\mathrm{M}},{\mathrm{atp}}}^{\mathrm{ACS}}$, affect the system dynamics, quantified with the largest eigenvalue of the Jacobian (Methods). To test this hypothesis, we split a population of 50,000 biologically relevant models into ten different subsets according to the $K_{{\mathrm{M}},{\mathrm{atp}}}^{\mathrm{ACS}}$ value, meaning that we had ten subpopulations of parameter sets with $K_{{\mathrm{M}},{\mathrm{atp}}}^{\mathrm{ACS}}$ ranging from low to high values (Fig. 3b, left). We computed the eigenvalue distribution for each of the ten subpopulations. As hypothesized, the mean eigenvalue becomes more negative as $K_{{\mathrm{M}},{\mathrm{atp}}}^{\mathrm{ACS}}$ increases (Fig. 3b, right), meaning that the models have faster dynamic responses for higher $K_{{\mathrm{M}},{\mathrm{atp}}}^{\mathrm{ACS}}$ values. This is also consistent with the $K_{{\mathrm{M}},{\mathrm{atp}}}^{\mathrm{ACS}}$ distributions showing that higher values of this parameter favour biological relevance (Fig. 3a). A similar analysis for all parameters in Fig. 3a showed no such trends for the last few parameters, confirming that these parameters are indeed sloppy (Supplementary Fig. 7a,b).

We repeated this study for the other three physiologies and obtained similar results (Supplementary Note 6). These results show that the GANs distil important information by learning the distributions of critical kinetic parameters and giving less importance to the parameters not affecting the desired property.

### Extrapolation to other physiologies using transfer learning

Comprehensive analyses of metabolic networks require large populations of parameter sets. However, a trade-off exists between generating large datasets and computational requirements, which can limit the scope of studies depending on the efficiency of the methods employed.

**Fig. 4:**
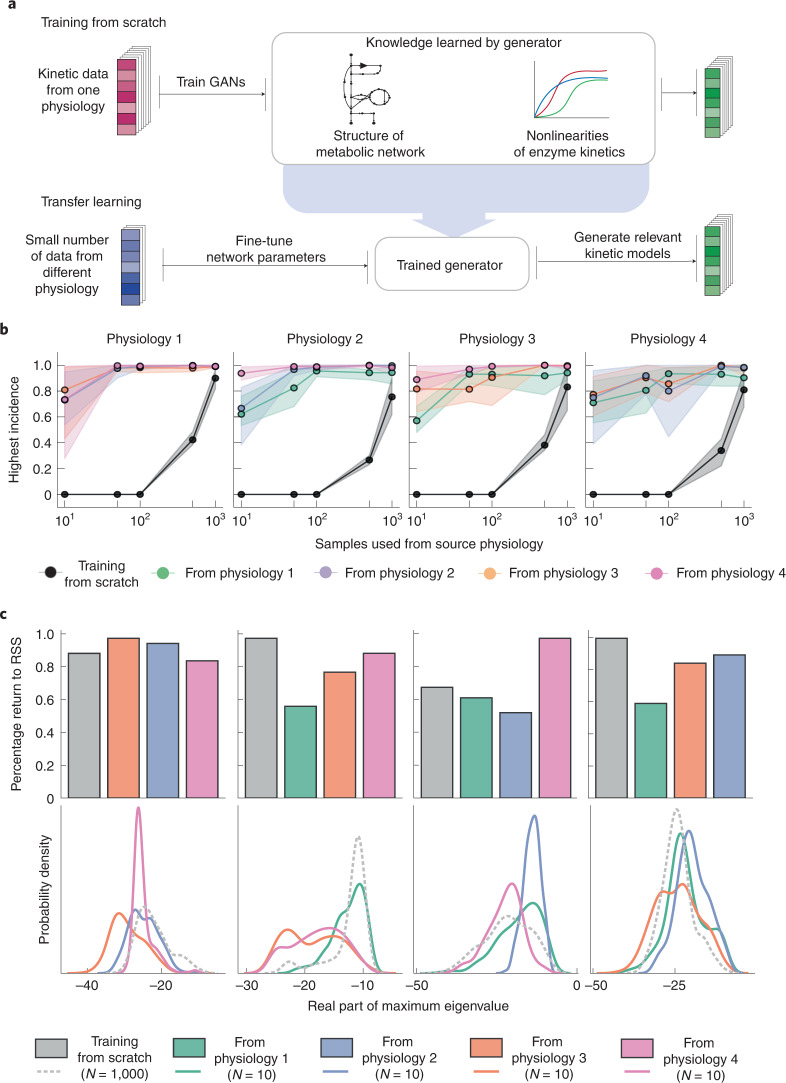
Extrapolation to multiple physiologies via transfer learning. **a**, A generator trained for one physiology learns the structure of the metabolic network and nonlinearities of enzymatic mechanisms, allowing us to retrain it for another physiology with just a few parameter sets. **b**, Comparison of the incidence of biologically relevant models created with the generators trained from scratch and with transfer learning for four physiologies. For each physiology, we compare the training from scratch and the transfer from the other three physiologies using different numbers of data (10, 50, 100, 500 and 1,000 datasets). **c**, Validation of transfer learning. Upper panel: fraction of the perturbed models that return to the reference steady state (RSS) for the models obtained from the generators trained from scratch and from the generators trained by transfer learning. Lower panel: probability density function of the real part of the maximum eigenvalue obtained for the populations of kinetic models obtained from the two types of generator trained from scratch and by transfer learning.

REKINDLE addresses this issue by leveraging the extrapolation abilities of GANs via transfer learning^41^: we fine tune a generator trained for one physiology for another physiology using a considerably smaller set of training models (Fig. 4a). To illustrate the benefits of this approach, we took the generator trained for physiology 1 and used it to retrain GANs for physiologies 2–4 using 10, 50, 100, 500 and 1,000 training kinetic models from the three target physiologies. For each physiology, we trained GANs for 300 epochs and repeated the training five times with a randomly weighed discriminator (Methods and Supplementary Fig. 11a–c). The transfer learning with only ten parameter sets provided a remarkably high incidence of biologically relevant models of physiologies 2–4 (Fig. 4c). Indeed, the incidence ranged from 72% (for the transfer from physiology 1 to physiologies 3 and 4) to 82% (physiology 1 to physiology 2). More strikingly, the transfer had already attained a very high incidence for all three physiologies with 50 parameter sets.

For comparison, we trained GANs from scratch for physiologies 2–4 using 10, 50, 100, 500 and 1,000 training parameter sets for 1,000 epochs in each training. Despite the shorter training (300 versus 1,000 epochs), the transfer learning considerably outperformed training from scratch (Fig. 4b). Training from scratch reaches a performance comparable to that of transfer learning only for around 1,000 parameter sets. Training from scratch completely fails when the number of samples is below 500, as the discriminator overpowers the generator^42^.

We also performed transfer learning from physiologies 2, 3 and 4 to the other three physiologies (for example, from physiology 2 to physiologies 1, 3 and 4). As expected, the transfer learning required considerably fewer data and training epochs compared with training from scratch (Fig. 4b). The transferred generators displayed good extrapolation results even when provided with ten models from the target physiology with the average incidence of biologically relevant models being between 0.6 and 0.8. The GANs for physiologies 1–3 trained by transfer learning from physiology 4 performed exceptionally well, with the incidence of feasible models reaching ~100% for 100 training sets (Fig. 4b, pink circles).

We further compared the features of the kinetic models obtained from the generators trained from scratch and those generated via transfer learning (Fig. 4c). Similarly to the study from Fig. 2e, we first investigated how many models evolve back to the reference steady state when their metabolic state is perturbed. The kinetic models generated via transfer learning had robustness properties similar to those from GANs trained from scratch despite using a much smaller dataset (ten parameter sets) (Fig. 4c). We then compared the kinetic parameter distribution of the two classes of models. A narrow parameter distribution could indicate that the generated models stem from a constrained region in the space and that generators are not producing diverse kinetic models. Instead of comparing the distributions of individual kinetic parameters, we used the distribution of the largest negative eigenvalue of the generated kinetic models as a meta-measure for the spread of parameter distributions (Fig. 4c). We observed that the models generated via transfer learning have a well spread distribution comparable to that of the models generated via learning from scratch.

We conclude that transfer learning successfully captures the specificities of the physiologies (Supplementary Note 7). With only a few kinetic parameter sets, transfer learning allows the generation of kinetic models that possess the desired properties of biological relevance, robustness and parametric diversity. We anticipate that this approach could help to derive new methods for high-throughput analysis of metabolic networks.

## Discussion and conclusions

The scarceness of experimentally verified information about intracellular metabolic fluxes, metabolite concentration and kinetic properties of enzymes leads to an underdetermined system with multiple models capable of capturing the experimental data. Due to the requirement of intense computational resources aiming to quantify the involved uncertainties, researchers often end up using only one out of the many alternative solutions, leading to unreliable analysis and misguided predictions about metabolic behaviour of cells. This is one of the reasons for the limited use of kinetic models in studies of metabolism, despite their widely acknowledged capabilities. REKINDLE offers a highly efficient way of sampling the parameter space and creating kinetic models, thus enabling an unprecedented level of comprehensiveness for analysing these networks and offering a much broader scope of applicability of kinetic models. In general, sampling of nonlinear parameter spaces has emerged as a standard method in addressing underdeterminedness in computational physics, biology and chemistry^43,44^.

The proof-of-concept applications presented here demonstrate REKINDLE’s ability to learn the mechanistic structure of the metabolic networks and stratify kinetic parameter subspaces corresponding to relevant model properties. By learning a map between the complex high-dimensional space of kinetic parameters and relevant model properties, the GANs augment (1) the efficiency of creating models corresponding to our specified criteria and (2) the information for partitioning the parameter space according to our criteria. Consequently, REKINDLE is several orders of magnitude faster than traditional methods in generating models. For GANs trained from scratch, REKINDLE requires ~1,000 data points to reliably reach a high incidence of relevant models (Fig. 4b), corresponding to a training time of ~15-20 min (Table 2). A trained REKINDLE generator generates 1 million models in ~18 s. In comparison, ORACLE, currently one of the most efficient kinetic modelling frameworks, accomplishes the same task in 18–24 h on the same hardware (Table 2). The reduction in generation time is even more pronounced when models are generated through transfer learning due to the small number of training data.

**Table 2:** Comparison of computational time between ORACLE, REKINDLE, and REKINDLE with transfer learning (REKINDLE-TL)

	Generation of training data	Training time	Generation of 1 million kinetic models	Generation of 1 million relevant kinetic models
ORACLE	—	—	~18–24 h	~36–48 h
REKINDLE	~15–20 min (1,000 models)	~15 min (1,000 models)	~15–20 s	~15–20 s
REKINDLE-TL	~5 s (10 models)	~2–3 min (10 models)	~15–20 s	~15–20 s

The generation time for 1 million biologically relevant kinetic models reduces by more than 6,000-fold when REKINDLE is used instead of ORACLE (last column). In total, when including the generation of training data and training time, REKINDLE and REKINDLE-TL are more efficient than ORACLE by more than 60- and 600-fold, respectively, in generating 1 million relevant models.

Once a generator is trained for the target physiology via transfer learning, it can be used to expand upon traditionally small datasets with newly generated synthetic datasets. Such expanded datasets are amenable for traditional statistical analyses to gain further knowledge about the studied system. This offers a crucial advantage to REKINDLE in scope of applications and comprehensiveness over traditional methods for generating kinetic models.

REKINDLE will allow construction of highly curated libraries of ‘off-the-shelf’ networks that have been pretrained using datasets from standard kinetic metabolic models. Such a repository will enable researchers to apply this framework to different physiologies and types of study and applications ranging from biotechnology to medicine.

In summary, we present a framework to generate kinetic models leveraging the power of deep learning while simultaneously retaining the convenience of traditional methods, where researchers can analyse the structural dependences, correlations and feedbacks within the metabolic network. The open-access code of REKINDLE will allow a broad population of experimentalists and modellers to couple this framework with experimental methods and benefit from synergistic approaches for the analysis and metabolic interventions of studied organisms.

## Methods

### Kinetic models of wild-type *E. coli*

Kinetic nonlinear models used in this study represent the central carbon metabolism of wild-type *E. coli*. They are based on the model published by Varma and Palsson^29^ and studied extensively using the SKimPy toolbox^35^ (Supplementary Fig. 1). The kinetic models consist of 64 metabolites, distributed over cytosol and extracellular space, and 65 reactions including 16 transport reactions. Out of the 64 metabolites in the model, 15 metabolites are boundary metabolites, that is, they are localized in the extracellular space. The remaining 49 metabolites are localized in the cytosol and their mass balances are described with the ODEs. After assigning each reaction to a kinetic mechanism on the basis of the stoichiometry, we parameterized this system of ODEs with 411 kinetic parameters.

### ORACLE framework and generation of the training set

The ORACLE framework consists of a set of computational procedures that allow us to build a population of large-scale kinetic models accounting for the uncertain and scarce information about the kinetic properties of enzymes^20,24,30–34,45^. The idea behind ORACLE is to reduce the feasible space of the kinetic parameters through the integration of available experimental measurements, omics data and thermodynamic constraints, and then employ Monte Carlo sampling techniques to determine unknown parameters^15,46^. ORACLE builds the kinetic models around the thermodynamically consistent reference steady-state fluxes and metabolite concentrations^30,47,48^. Instead of directly sampling the kinetic parameters such as Michaelis–Menten constant and inhibitory constants, we sample the enzyme saturation in the following form^41^:

$$\sigma _{ij} = \frac{{\left[ {S_i} \right]/K_{\mathrm{M}}^{ij}}}{{1 + \left[ {S_i} \right]/K_{\mathrm{M}}^{ij}}}.$$

Then, we back-calculate the values for the kinetic parameters, $K_{\mathrm{M}}^{ij}$, using the knowledge of the steady-state concentrations, [*S*_*i*_], and the enzyme saturation, *σ*_*ij*_. Once we know the $K_{\mathrm{M}}^{ij}$, the equilibrium constants $K_{\mathrm{eq}}^j$ obtained when calculating the thermodynamically consistent steady state and the steady-state flux *v*_*j*_, we can calculate the maximal velocities, $V_{\mathrm{max}}^{j}$, by substituting these quantities in the rate expressions. This way, the kinetic model is completely parameterized.

### Determining biological relevance and dataset labelling

We consider the kinetic models biologically relevant if these models are locally stable and all characteristic times of the aperiodic model response fall within physical and biological limits. To test the local stability and time constants of the generated models, we compute the Jacobian of the dynamic system^46^. The sign of the eigenvalues of the Jacobian gives us information on whether or not the generated models are locally stable. If the real parts of all eigenvalues are negative for a model, then the model is locally stable. Otherwise, if any real part of the eigenvalues is positive, the model is unstable.

Moreover, for a locally stable system, we define the characteristic time constants of the linearized system as the inverse of the real part of the largest eigenvalue of the Jacobian. The characteristic time constants allow us to characterize the model dynamics. Small time constants emerge from fast metabolic processes such as electron transport chain and glycolysis, whereas the slower timescale emerges from biosynthetic processes. Physical and biological limits bind all these timescales.

Therefore, we consider that the aperiodic model response should not exceed the timescale of cell division. We enforce this constraint by considering that all characteristic response times should be three times faster than the cell’s doubling time, ensuring that a perturbation of the metabolic processes settles within 5% of the operating steady state before the subsequent cell division. One might further consider other constraints on the response dynamics: for example, that the biochemical response should exhibit a characteristic time slower than the timescale of proton diffusion within the cell. Models satisfying these properties can reliably capture the metabolic responses observed in nature.

In this study, the dynamic responses of our models should be at least three times faster than *E. coli’s* doubling time (~21 min)^36^, that is, the dominant time constant of models’ responses should be smaller than ~7 min. Therefore, we impose a strict upper bound of Re(*λ*_*i*_) < −9 (~−60/7) on the real part of the eigenvalues, *λ*_*i*_, of the Jacobian. We label all kinetic parameter sets that obey this constraint as biologically relevant; otherwise, they are labelled irrelevant.

### Data preprocessing

After labelling, the dataset was log transformed, as the kinetic parameters spanned several orders of magnitude. The generated dataset of 80,000 models was then split into training and test sets with the ratio 9:1, which resulted in 72,000 models that were used for training the conditional GAN. Moreover, only the concentration-associated parameters, $K_{\mathrm{M}}^{ij}$, were included as the training features, because the scaling coefficients, $V_{\mathrm{max}}^{j}$, can be calculated back once the $K_{\mathrm{M}}^{ij}$ and steady-state concentration and flux profiles are already known. After eliminating $V_{\mathrm{max}}^{j}$, the training dataset consisted of 259 features in total.

It should be noted that both classes of models in the training data, biologically feasible and infeasible, have statistically large overlap in the kinetic parameter space and cannot be independently visualized by low-order dimension reduction techniques such as PCA^49^, *t*-distributed stochastic neighbour embedding^50^ or Uniform Manifold Approximation and Projection^51^ (Supplementary Fig. 9).

During the training, the KL divergence between the test dataset and the GAN-generated dataset was also monitored as an additional step to verify that training was successful (Supplementary Note 4).

### Perturbation analysis of kinetic models

We randomly choose 1,000 biologically relevant kinetic parameter sets from both the ORACLE-sampled dataset and the REKINDLE-generated dataset for any given physiology. We parameterize the system of ODEs describing the metabolite concentrations using REKINDLE- or ORACLE-generated kinetic parameter sets. Then, for each parameterized system we randomly perturb the reference steady-state metabolite concentration (*X*_ref_) and flux profile of the model up to ±50%. We next integrate the ODEs using this perturbed state *X*′ as the initial condition *X*(*t* = 0) = *X*_0_. To quantify whether a model has returned to the reference steady state, we monitor the *L*_2_ norm of the metabolite concentrations at a given point of time *X*(*t*) and the reference concentration *X*_0_. If the metabolite concentration at 21 min (doubling time of *E. coli*) is less than 1% of the reference steady state we classify the model as having returned to the steady state, that is, we test

$$\left| X\left( t \right)_{t = 21\,{\mathrm{min}}} - X_{\mathrm{ref}}\right| < 0.01\left|X_{\mathrm{ref}}\right|.$$

We repeat this process ten times for each parameter set with a random perturbation each time (within ±50%).

### KL divergence/relative entropy

For two separate probability distributions *P*(*x*) and *Q*(*x*) over the same random variable *x*, we can measure how different these distributions are using the KL divergence^52^ from *Q*(*x*) to *P*(*x*), formulated as

$$D_{\mathrm{KL}}(P||Q) = \left\{ {\begin{array}{*{20}{c}} {{{\Sigma }}P\left( x \right){\mathrm{log}}\left( {\frac{{P(x)}}{{Q(x)}}} \right)} & {{{{\mathrm{if}}}}\;P\left( x \right) \ne Q(x)} \\ 0 & {{{{\mathrm{if}}}}\;P\left( x \right) = Q(x)} \end{array}} \right. .$$

### Spearman correlation coefficient

For two random variables *X* and *Y*, we compute the Spearman correlation coefficient as

$$\rho = \frac{{{\mathrm{cov}}(R\left( X \right),R(Y))}}{{\sigma _{R\left( X \right)}\sigma _{R\left( Y \right)}}}$$

where *R*(*X*) and *R*(*Y*) are the ranks of *X* and *Y*, respectively, cov(*R*(*X*), *R*(*Y*)) the covariance of the rank variables and *σ*_*R*(*X*)_ and *σ*_*R*(*Y*)_ are the s.d. of the rank variables.

### GAN training

In GANs, two neural networks, the generator that we train to generate new data and the discriminator that tries to distinguish generated new data from real data, are pitted against each other in a zero-sum game. The end goal of this game is to obtain the generator that generates new data of such a quality that the discriminator cannot distinguish it from real data (Fig. 1a, step 3). We train the generator and discriminator networks in turn. To train the discriminator, we freeze the generator by fixing its network weights. Then, we alternate the training by freezing the discriminator and train the generator. In the first part of each learning step, we provide the discriminator with (1) a random batch of kinetic parameter sets from the training data with labels indicating the class of models and (2) a batch of kinetic parameter sets that have been generated by the generator (fake data). The discriminator then classifies the models it is presented with as real (from the training set) or fake (from the generator). In the second part of a learning step, the discriminator is frozen and the generator generates a batch of fake kinetic parameter sets using as inputs (1) random Gaussian noise and (2) sampled labels. The discriminator and the generator improve their performance with training. The generator becomes better at deceiving the discriminator, and the discriminator becomes better at classifying between training and generated data (Fig. 1a, step 2). The training continues until we reach equilibrium between the two neural networks, and no further improvement is possible.

The minimum number of data required to train a randomly initialized GAN for generating relevant models depends on the sizes of the neural networks used and the metabolic system studied. Determining the minimal number of data for training as a function of the size of the studied metabolic system remains an open problem.

### GAN implementation

All software programs were implemented in Python (v3.8.3) using Keras (https://keras.io/, v2.4.3) with a TensorFlow^53^ graphics processing unit (GPU) backend (www.tensorflow.org, v2.3.0). The GANs were implemented as conditional GANs. The discriminator network was composed of three layers that have a total of 18,319 parameters: layer 1, Dense with 32 units, Dropout (0.5); layer 2, Dense with 64 units, Dropout (0.5); layer 3, Dense with 128 units, Dropout (0.5). The generator network was composed of three layers that have a total of 315,779 parameters: layer 1, Dense with 128 units, BatchNormalization, Dropout (0.5); layer 2, Dense with 256 units, BatchNormalization, Dropout (0.5); layer 3, Dense with 512 units, BatchNormalization, Dropout (0.5). We used the binary cross-entropy loss and the Adam optimizer^54^ with a learning rate of 0.0002 for training both the networks. We used batch sizes of 2, 5, 10, 20 and 50 when training with training set sizes of 10, 50, 100, 500 and 1,000, respectively. We trained the GANs from scratch over each physiology for 1,000 epochs (one epoch is defined as one pass over all of the training data), which took approximately 40 min for training over 72,000 models on a single Nvidia Titan XP GPU with 12 GB of memory. Training was repeated five times for each physiological condition with randomly initialized generator and discriminator networks (20 total trainings).

In all studies, we consider that training has failed if the discriminator accuracy is consistently greater than 90% for the last 200 epochs. In some cases, extending training to 1,500 epochs was necessary to maximize the incidence of desired models. Training beyond 1,500 epochs showed negligible increase in performance. In some cases, training for too many epochs led to GAN collapse, that is, the discriminator overpowered the generator.

Finding the optimum architecture of the REKINDLE neural networks relative to the size of the studied metabolic network remains an open problem.

### Generation of biologically irrelevant kinetic models

To study the statistical differences in the distributions of the biologically relevant and non-relevant kinetic models we generated two populations (2,000 models) of each class using a trained generator. As most of the trained generators do not have 100% incidence of biologically relevant models (Table 1), they have a small incidence of non-relevant kinetic models even when conditionally generating relevant models. We use this to create a population of non-relevant kinetic models, by continuously generating relevant models using the respective conditional label until we obtain a population of 2,000 non-relevant models. Alternatively, we can also generate non-relevant kinetic models by directly using the appropriate conditional label with the generator seed during generation. However, when we subjected the population generated in this manner to statistical analysis (calculating the KL divergences between the individual kinetic parameters) we failed to retrieve the important parameters. We hypothesize that this is due to the absence of important kinetic parameters that determine the local instability of a kinetic model (Supplementary Note 5).

### Transfer learning on multiple physiologies

We trained GANs from scratch for each physiology using 72,000 samples, and saved the generator state for the generator that had the highest incidence of relevant models. Then we retrained this generator in a GAN setting with a randomly weighted discriminator using a small number of data (10, 50, 100, 500 and 1,000) from the target physiology on which extrapolation is desired, for 300 epochs. For the instances using 500 and 1,000 samples the learning rate was changed from 0.0002 to 0.001. For the rest of the cases, we trained with the same hyperparameters as discussed in the previous section. During training, we generated 1,000 biologically relevant models every 10 epochs and calculated the eigenvalues of the Jacobian, as previously, to monitor the quality of training and the ability of the generator to generate biologically relevant models from the target physiology. We repeated the training five times for each transfer (physiology a to physiology b) with the same pretrained generator (on physiology a) but with a randomly weighted discriminator each time. We noted the highest incidence of relevant models during training save the corresponding generator state for each instance of training. We repeated this entire procedure (transferring generator, retraining GAN, generation of relevant models and validation using eigenvalues of Jacobian) for different numbers of data from the target physiologies (10, 50, 100, 500 and 1,000 samples). Total training counts: 4 (total physiologies) × 3 (target physiologies) × 5 (repeats) × 5 (number of data used) = 300. The resulting highest incidence of biologically relevant models from the target physiology as a function of the number of data samples used is summarized in Fig. 4b.

### Reporting summary

Further information on research design is available in the Nature Research Reporting Summary linked to this article.

### Supplementary information

Supplementary Information Supplementary Figs. 1–19, Tables 1 and 2 and Notes 1–8.

Reporting Summary

## Data Availability

The data that support the findings of this study are publicly available in the Zenodo repository (https://zenodo.org/record/5803120 and the links therein).

## Code availability

A TensorFlow implementation of the REKINDLE workflow is publicly available at https://github.com/EPFL-LCSB/rekindle and https://gitlab.com/EPFL-LCSB/rekindle (ref. ^55^). The ORACLE framework is implemented in the SKiMpy (Symbolic Kinetic Models in Python)^35^ toolbox, available at https://github.com/EPFL-LCSB/skimpy.
