# Supplementary material for: Reconstructing Kinetic Models for Dynamical Studies of Metabolism using Generative Adversarial Networks
Source: Nat Mach Intell. 2022 Aug 30;4(8):710–9. doi: 10.1038/s42256-022-00519-y (PMC10543203; doi:10.1038/s42256-022-00519-y)
Supplement: Supplementary file 1 — Supplementary Figs. 1–19, Tables 1 and 2 and Notes 1–8. [file 42256_2022_519_MOESM1_ESM.pdf]

nature portfolio

https://doi.org/10.1038/s42256-022-00519-y

Supplementary information

Reconstructing Kinetic Models for Dynamical Studies of Metabolism using Generative Adversarial Networks

In the format provided by the authors and unedited

Supplementary information

Reconstructing Kinetic Models for Dynamical Studies of Metabolism using Generative Adversarial Networks

Subham Choudhury[1], Michael Moret[1], Pierre Salvy[1,2], Daniel Weilandt[1,3], Vassily Hatzimanikatis[1*], Ljubisa Miskovic[1*]

[1] Laboratory of Computational Systems Biology (LCSB), Ecole Polytechnique Fédérale de Lausanne (EPFL), CH-1015 Lausanne, Switzerland

[2] Present address: Cambrium GmBH. Berlin, Germany

[3] Present address: Princeton University, Princeton, USA

Correspondence: ljubisa.miskovic@epfl.ch, vassily.hatzimanikatis@epfl.ch

Content

Supplementary figures

Supplementary Figure 1: Escher map of the E. coli central carbon metabolism network.

Supplementary Figure 2: Discriminator loss, generator loss and discriminator accuracy when training from scratch for (a) Physiology 1 (b) Physiology 2 (c) Physiology 3 (d) Physiology 4

Supplementary Figure 3: Generation and validation of REKINDLE generated datasets for (a) Physiology 2 (b) Physiology 3 (c) Physiology 4

Supplementary Figure 4: Perturbation analysis of REKINDLE generated datasets for (a) Physiology 2 (b) Physiology 3 (c) Physiology 4

Supplementary Figure 5: KL divergence scores between the distributions of individual kinetic parameters in the relevant and non-relevant classes of REKINDLE generated kinetic parameter sets for Physiology 1

Supplementary Figure 6: The top 7 kinetic parameters from Fig 3a are visualized in the metabolic network.

Supplementary Figure 7: Distribution of the eigenvalues of the Jacobian of subpopulations based on the values of the 2 lowest ranked parameters in Fig 3a.

Supplementary Figure 8: Highest ranked parameters based on KL divergence scores between relevant and non-relevant REKINDLE generated kinetic parameter sets, the respective metabolic subsystems they belong to and the effect of constraining them for Physiology 2, 3 and 4.

Supplementary Figure 9: Visualization of the kinetic parameter space of the training data for Physiology 1 using (a) PCA (b) UMAP (c) tSNE.

Supplementary Figure 10: Visualization of the kinetic parameter space of the training data and REKINDLE generated data for Physiology 1 using UMAP (a) for all the kinetic parameters (b) top 5 parameters from Fig 3a.

Supplementary Figure 11: (a) Discriminator loss and generator loss for transfer learning from Physiology 4 to 1 using 10, 50, 100, 500 and 1000 samples from Physiology 1. (b) Discriminator Accuracy for the same specifications as (a) (c) Incidence of biologically relevant models for the same specifications as (a).

Supplementary Figure 12: Classification accuracies of standard machine learning models between biologically relevant and irrelevant kinetic parameter sets for physiology 1.

Supplementary Figure 13: Kullback Leibler divergence between the REKINDLE generated data and (i) training data (solid light green) (ii) test data (solid teal line) for Physiology 1 for 5 statistical repeats.

Supplementary Figure 14: Kullback Leibler divergence between the REKINDLE generated data and (i) training data (ii) test data (solid teal line) (iii) control data (black-brown line) for Physiology 1 for 5 statistical repeats.

Supplementary Figure 15: Differences in distributions of individual kinetic parameters (y-axis) using KL divergence between 16 discretized subpopulations based on eigenvalue space (x-axis) near the class boundary i.e., $\mathrm{Re}(\lambda_{\max}) = -9$ for (a) Physiology 1 (b) Physiology 2 (c) Physiology 3 (d) Physiology 4.

Supplementary Figure 16: Differences in distributions of individual kinetic parameters (y-axis) using KL divergence between 16 discretized subpopulations based on eigenvalue space (x-axis) near the class boundary i.e., $\mathrm{Re}(\lambda_{\max}) = -9$ for (a) Physiology 1 (b) Physiology 2 (c) Physiology 3 (d) Physiology 4. Here the KL divergence for each sub-population is normalized between 0 and 1.

Supplementary Figure 17: (Left) The parameters in Fig 3a (main manuscript) are constrained within 90-th percentile and 100-th percentile (dashed vertical lines) of the relevant class (green) in the direction that favors biological relevancy. **(Right)** The fraction of locally unstable, stable and relevant models as Fig 3a result of constraining the parameters as explained in the left panel. We begin with 100 locally unstable models (orange bar, left) and gradually constrain the parameters in a cumulative manner, based on their ranks (right). Constraining the parameters stabilizes (blue) as well as rescues (green) the unstable models. We notice that there is a negligible change in dynamics after the top 7 parameters have been constrained.

Supplementary Figure 18: The KL divergences between distributions of 259 individual kinetic parameters, parameterized by ORACLE, among the 4 physiologies used.

Supplementary Figure 19: Distribution of maximum eigenvalues of relevant generated kinetic models (purple violins) over the course of training (left to right) for a) Physiology 1, b) Physiology 2, c) Physiology 3, d) Physiology 4, compared to the training and test data (orange violin, both distributions overlap). The dashed lines indicated the maximums and minimums of the test and training datasets.

Supplementary tables

Supplementary Table 1: The reaction and the rate law formalisms of the top 7 kinetic parameters from Fig. 3a.

Supplementary Table 2: Incidence of biologically relevant and non-relevant models generated with REKINDLE for four physiologies.

Supplementary notes

Supplementary Note 1: Testing classification performance of different machine learning algorithms on kinetic parameter sets.

Supplementary Note 2: Advantages of REKINDLE over traditional sampling-based kinetic modeling methods.

Supplementary Note 3: Checking for overfitting in GANs

Supplementary Note 4: Validating statistical similarity of generated data against test dataset and randomized dataset.

Supplementary Note 5: Generation of biologically irrelevant models using REKINDLE.

Supplementary Note 6: Interpretability of REKINDLE results

Supplementary Note 7: Differences in kinetic parameter distributions between different physiologies.

Supplementary Note 8: Extrapolation beyond test and training datasets.

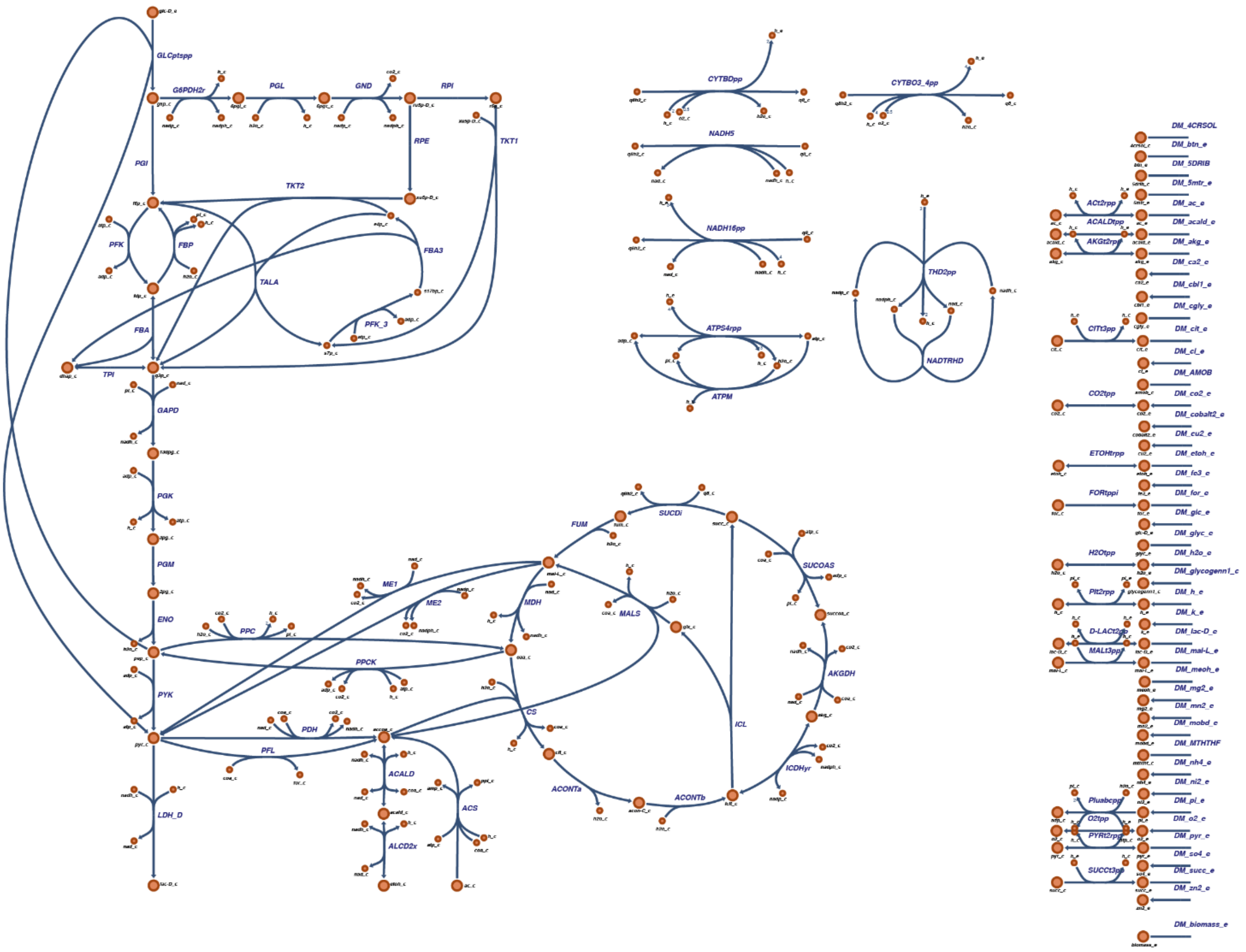

Supplementary Figure 1: Escher map of the *E. coli* central carbon metabolism network. The reactions are represented as blue lines and the metabolites as orange circles. This map was generated using the Escher web tool[1].

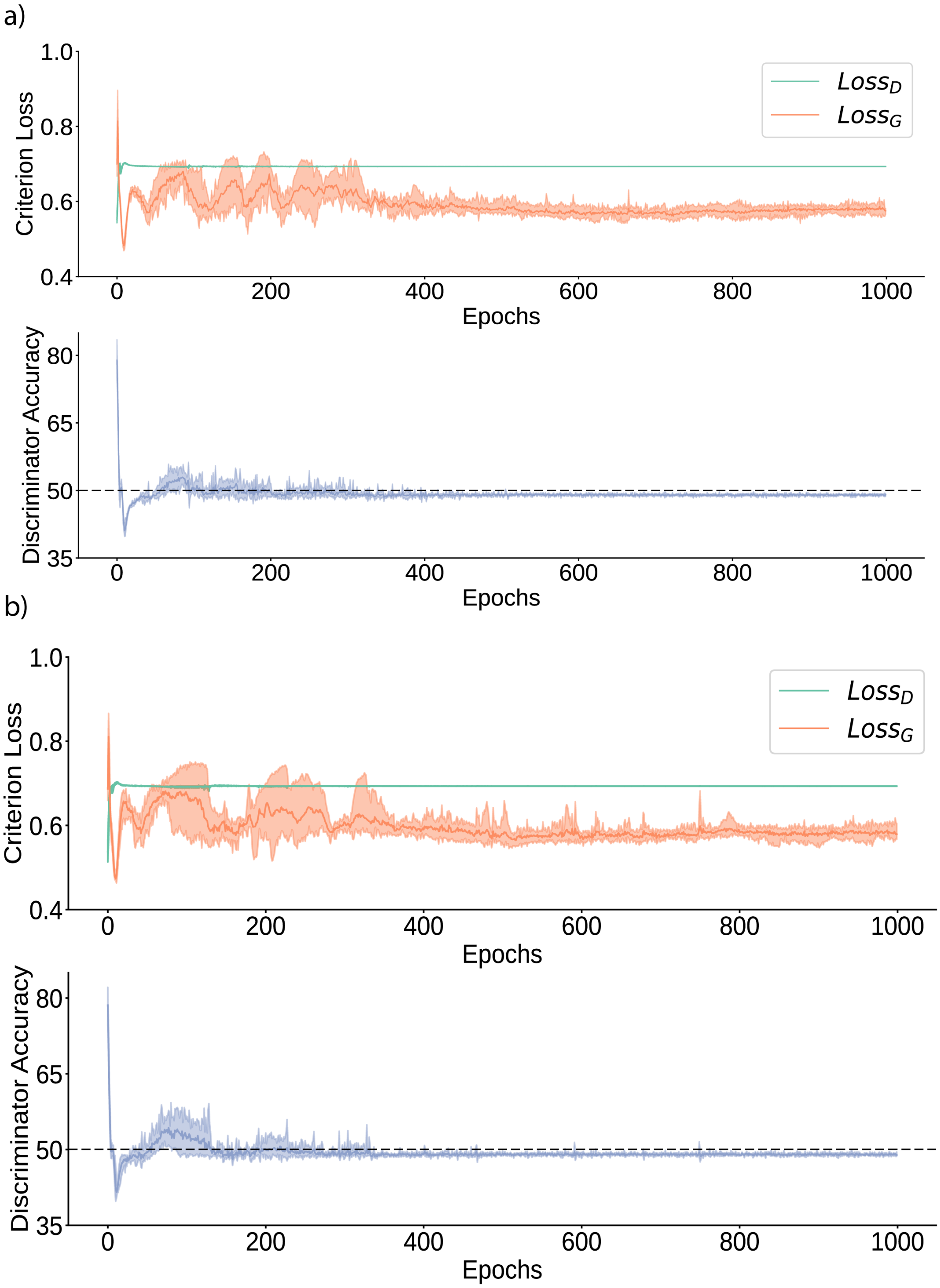

c)

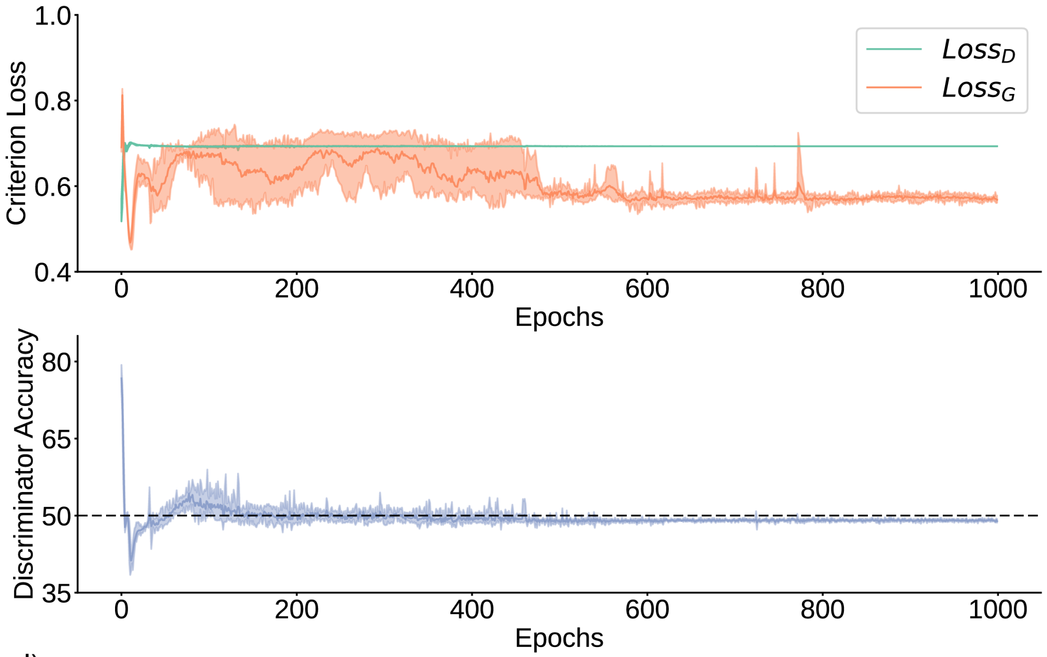

d)

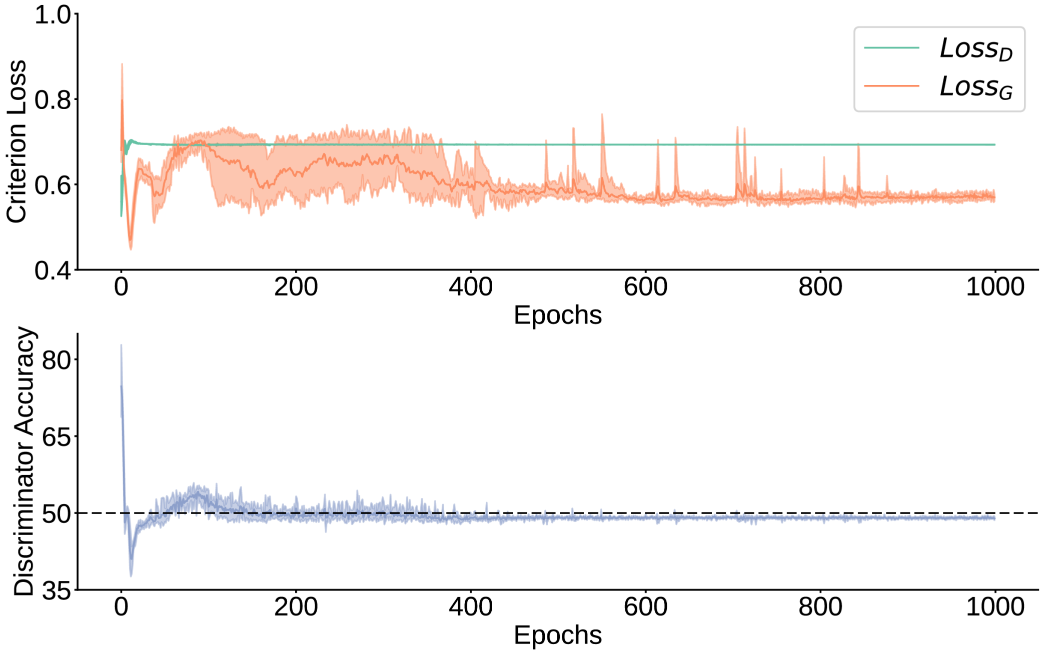

Supplementary Figure 2: Upper Panel: Discriminator Loss (green) and generator Loss (orange) values over training epochs for 5 statistical repeats. Lower Panel: Discriminator Accuracy over training epochs (5 statistical repeats) for (a) Physiology 1 (b) Physiology 2 (c) Physiology 3 (d) Physiology 4. The losses of the discriminator and generator stabilize as training progresses. The discriminator accuracy settles near 50% indicating that the discriminator cannot distinguish between training data (from ORACLE) and generated data (from the generator).

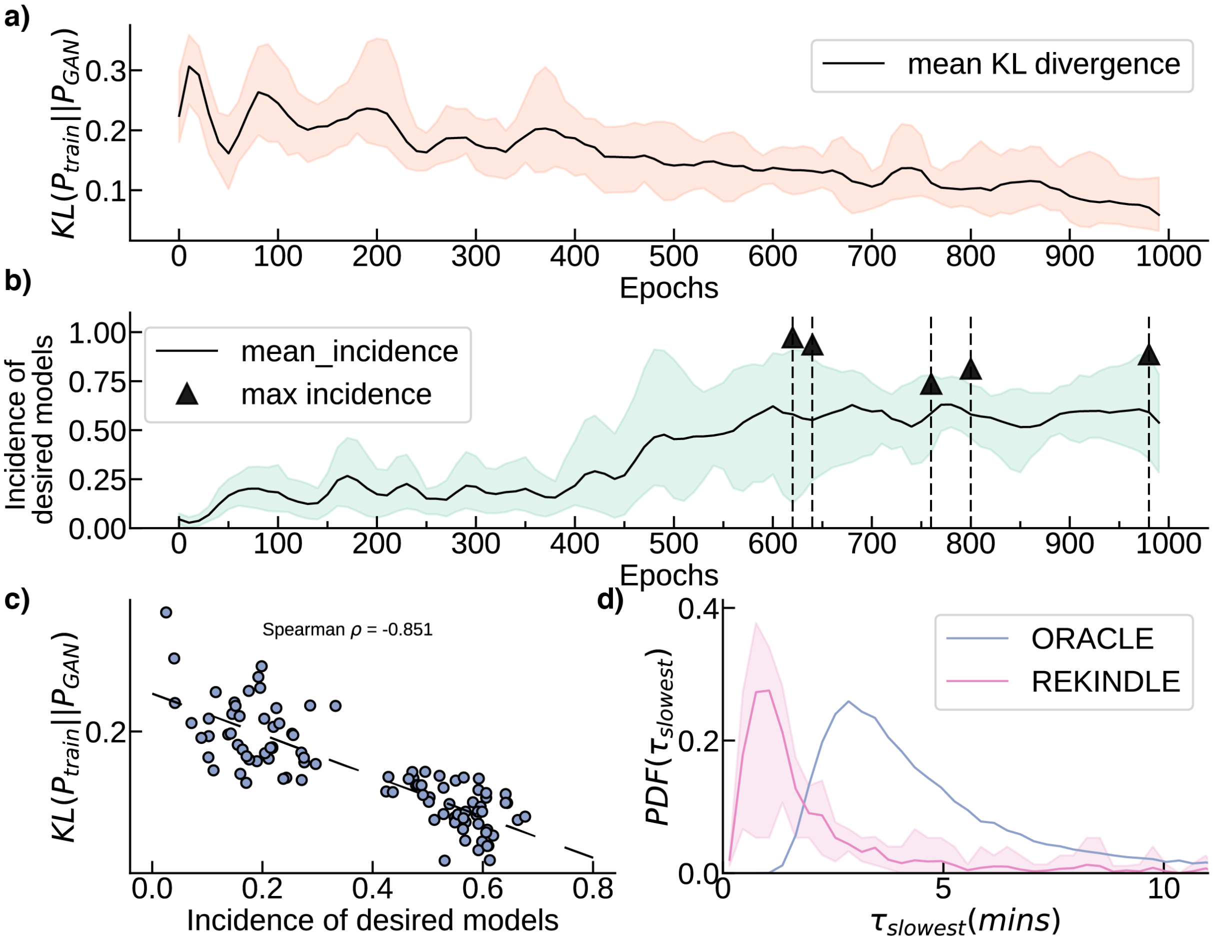

Supplementary Figure 3a: Generation and Validation of REKINDLE generated datasets for Physiology 2. The panels description and the meaning of abbreviations are presented in the caption of Figure 2 of the main manuscript.

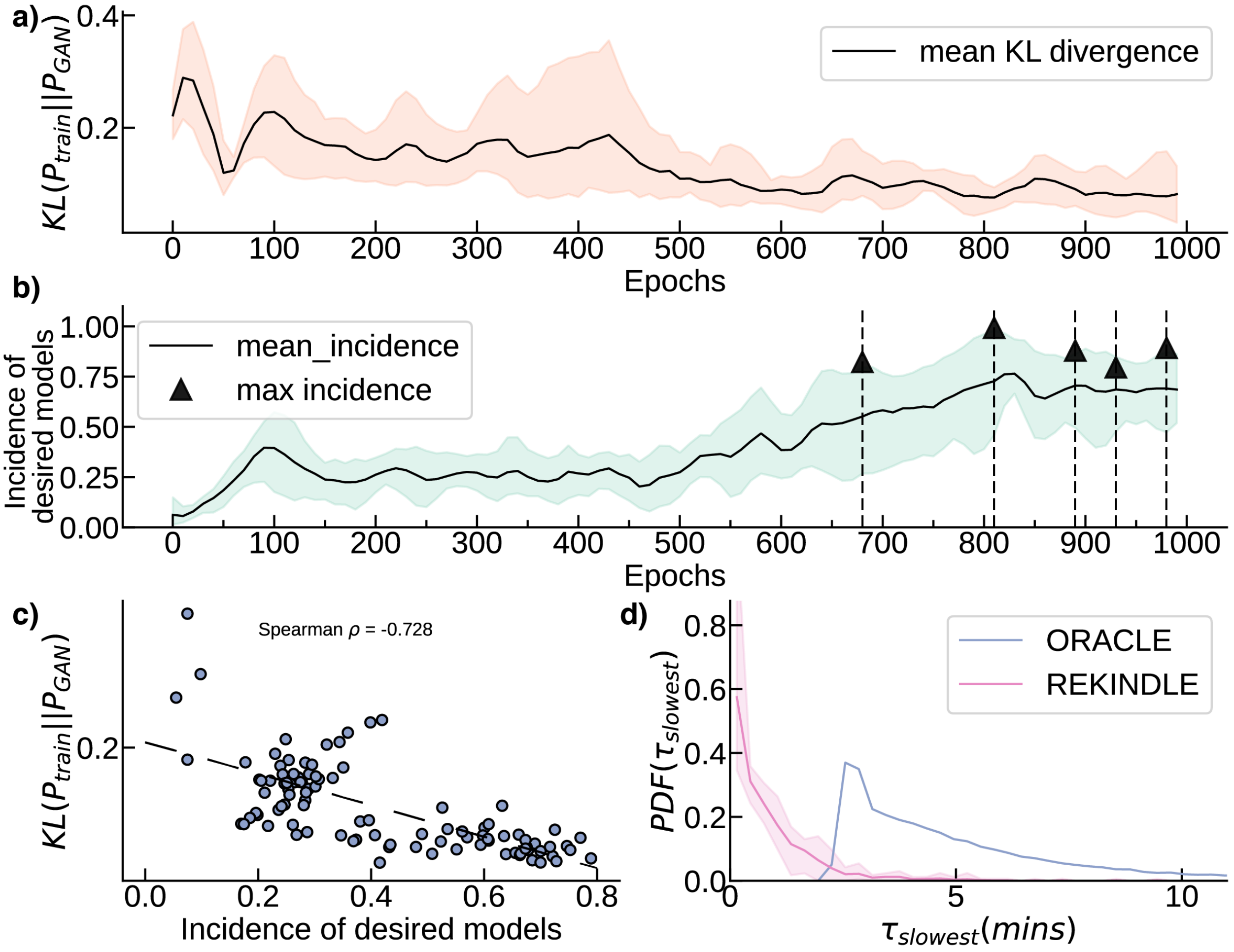

Supplementary Figure 3b: Generation and Validation of REKINDLE generated datasets for Physiology 3. The panels description and the meaning of abbreviations are presented in the caption of Figure 2 of the main manuscript.

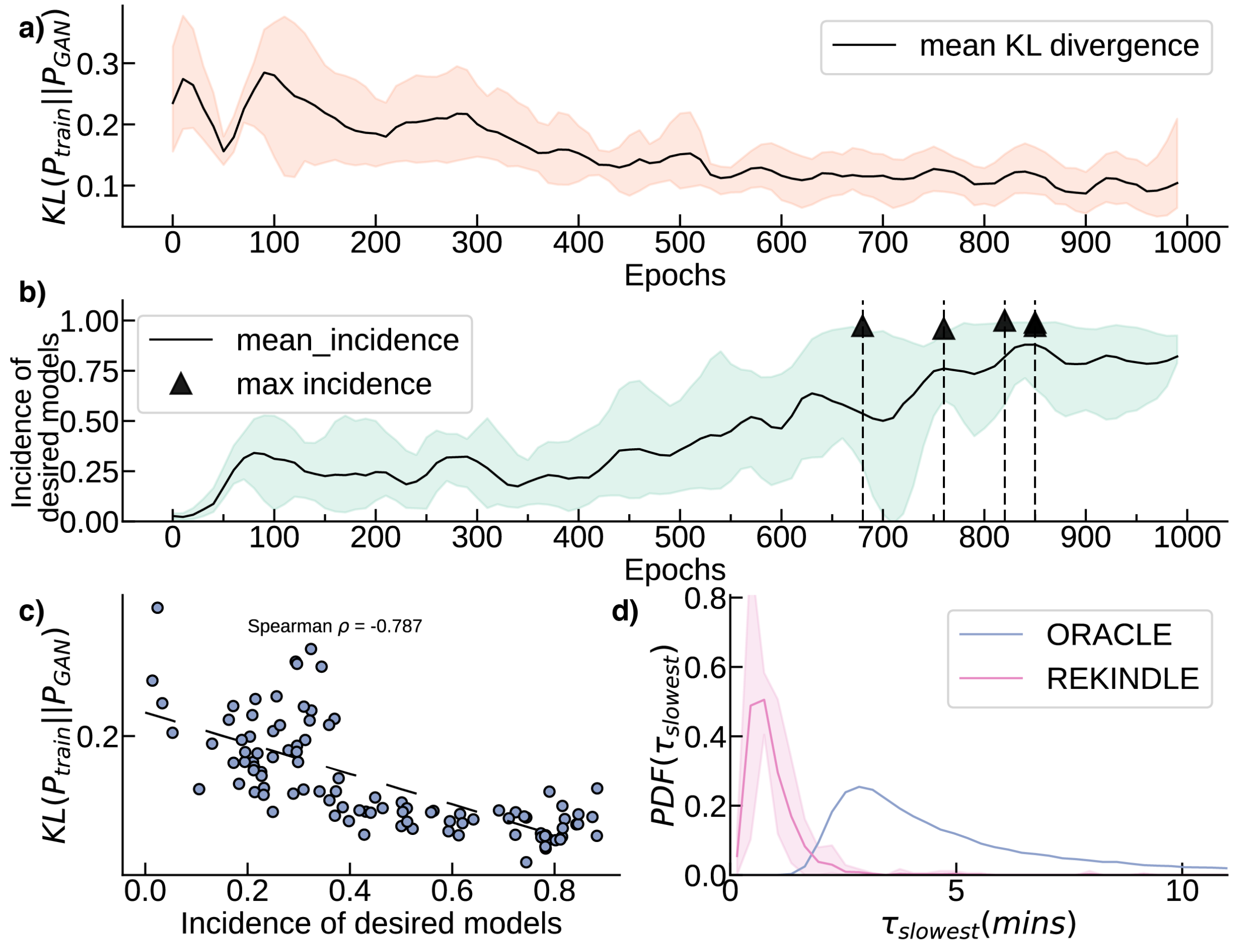

Supplementary Figure 3c: Generation and Validation of REKINDLE generated datasets for Physiology 4. The panels description and the meaning of abbreviations are presented in the caption of Figure 2 of the main manuscript.

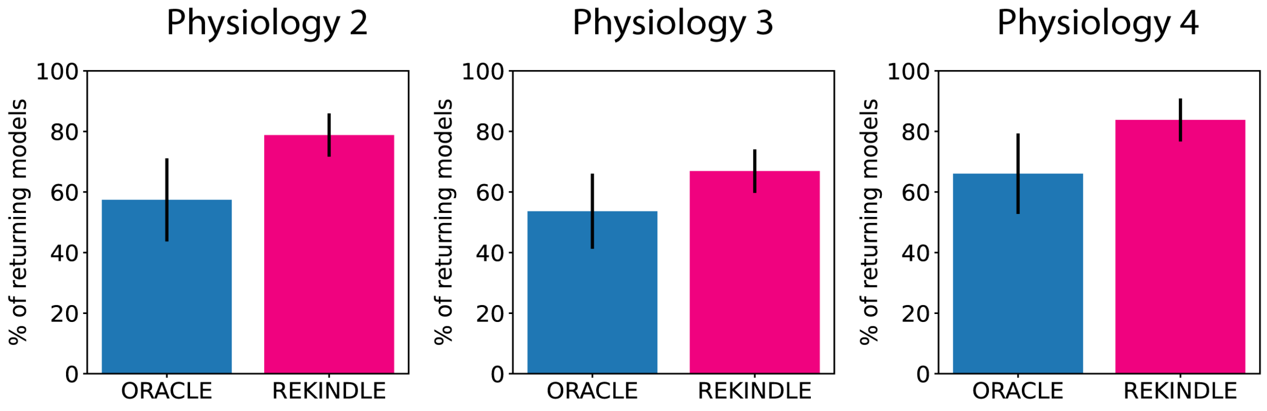

Supplementary Figure 4: 1000 models were perturbed 10 times randomly between $0.5X_{RSS} \leq \Delta X \leq 2X_{RSS}$ and then allowed to evolve with the perturbed state, ΔX, as the initial condition and then checked if the dynamics comes back to within 1% of the reference steady state for (a) Physiology 2 (b) Physiology 3 (c) Physiology 4. We see that higher percentage of REKINDLE parameterized kinetic models comes back to the steady state compared to ORACLE.

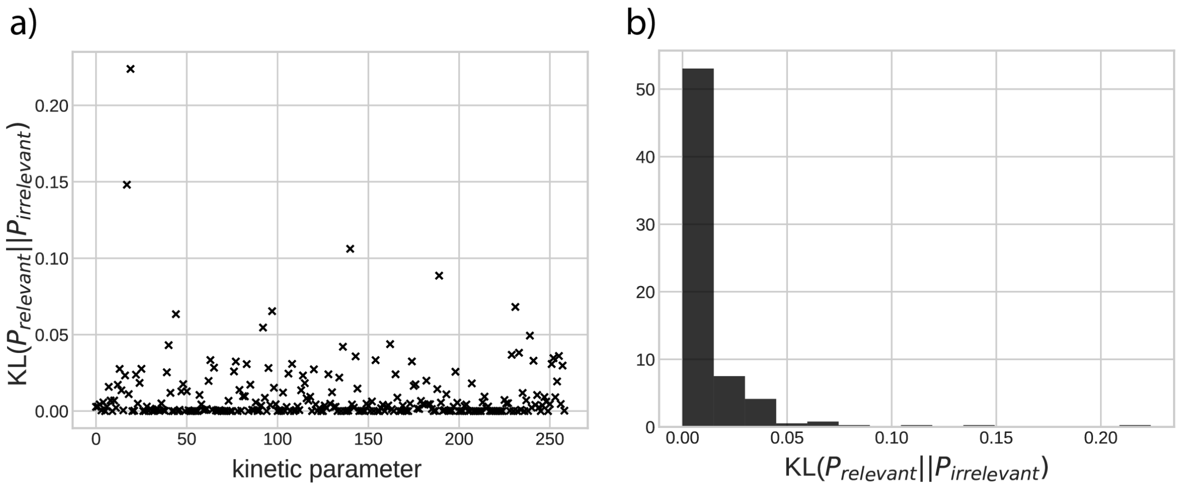

Supplementary Figure 5: (a) KL divergence scores of kinetic parameters distributions with biologically relevant and non relevant dynamics for Physiology 1. (b) Distributions of the KL divergence scores in (a). We observe that only a handful of parameters have significant KL divergence scores and majority of the parameters have KL divergence score close to zero. This indicates the most parameters have the same distributions in the kinetic models with relevant and non-relevant dynamics.

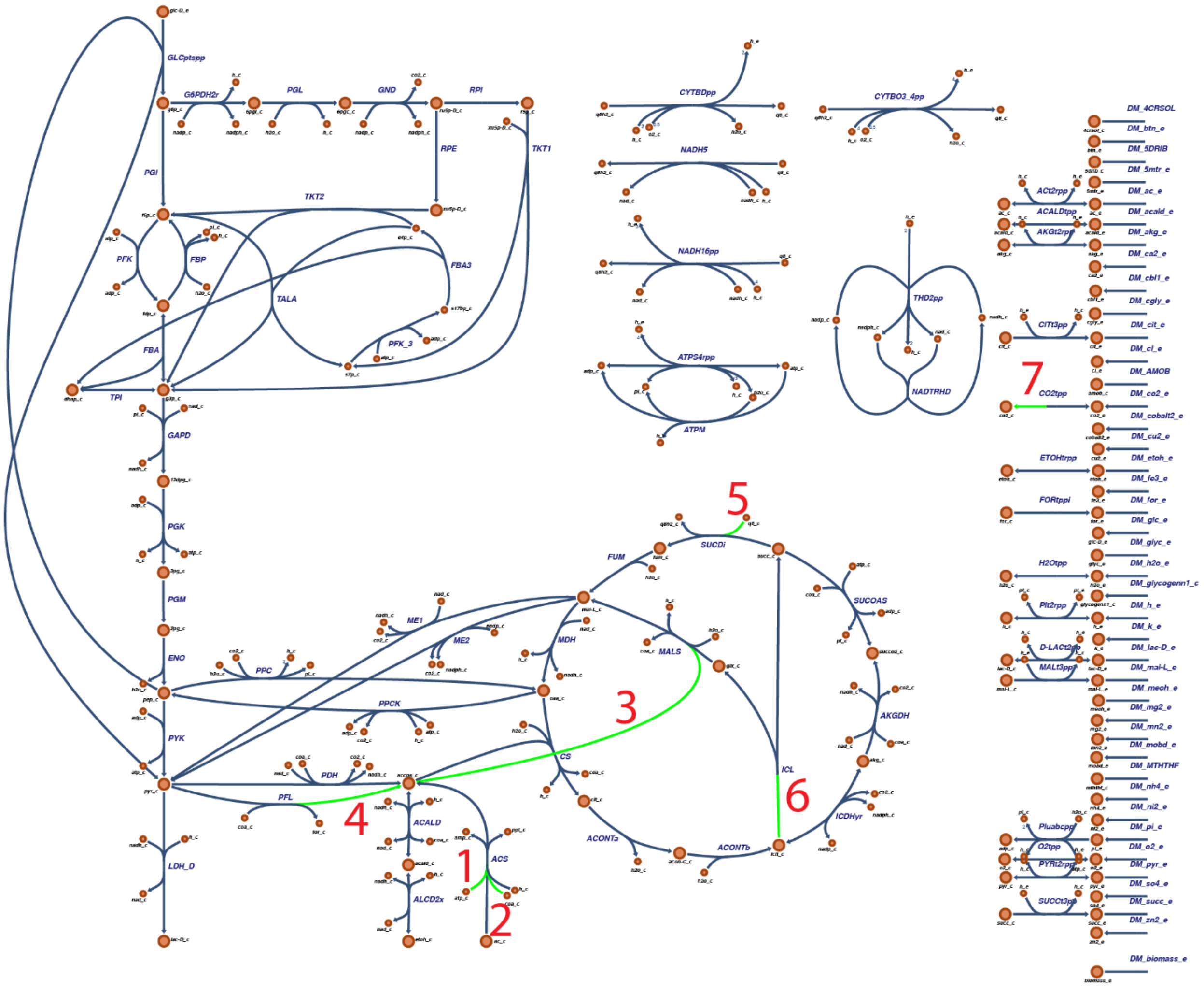

Supplementary Figure 6: The top 7 kinetic parameters from Fig 3a are visualized in the metabolic network. The green highlight indicates the substrate of the kinetic parameter with their associated reaction. The red numbers indicate the rank of the kinetic parameter according to Figure 3a. The exact reactions and rate law expressions of these 7 parameters are elaborated in Supplementary Table 1.

Kinetic Parameter	Reaction Name, Reaction	Rate law repression
Rank 1, $K^{ACS}_{M,atp}$	ACS: $coa_c + ac_c + atp_c \rightleftharpoons accoa_c + ppi_c + amp_c$	$v = \frac{V_{max}\left(1-\frac{1}{K_{eq}}\prod_{i=1}^{N}\left(\frac{[P_i]}{[S_i]}\right)\right)\prod_{i=1}^{N}\left(\frac{[S_i]}{K_{M,S_i}}\right)\left(\frac{[S_i]}{K_{M,S_i}}+\frac{[P_i]}{K_{M,P_i}}\right)^{h-1}}{\prod_{i=1}^{N}\left(1+\left(\frac{[S_i]}{K_{M,S_i}}+\frac{[P_i]}{K_{M,P_i}}\right)^{h}\right)}$ Parameters: $[V_{max}, K^{ACS}_{M,coa}, K^{ACS}_{M,ac}, K^{ACS}_{M,atp}, K^{ACS}_{M,accoa}, K^{ACS}_{M,ppi}, K^{ACS}_{M,amp}, K_{eq}, h, N = 3]$
Rank 2, $K^{ACS}_{M,coa}$	ACS: $coa_c + ac_c + atp_c \rightleftharpoons accoa_c + ppi_c + amp_c$	$v = \frac{V_{max}\left(1-\frac{1}{K_{eq}}\prod_{i=1}^{N}\left(\frac{[P_i]}{[S_i]}\right)\right)\prod_{i=1}^{N}\left(\frac{[S_i]}{K_{M,S_i}}\right)\left(\frac{[S_i]}{K_{M,S_i}}+\frac{[P_i]}{K_{M,P_i}}\right)^{h-1}}{\prod_{i=1}^{N}\left(1+\left(\frac{[S_i]}{K_{M,S_i}}+\frac{[P_i]}{K_{M,P_i}}\right)^{h}\right)}$ Parameters: $[V_{max}, K^{ACS}_{M,coa}, K^{ACS}_{M,ac}, K^{ACS}_{M,atp}, K^{ACS}_{M,accoa}, K^{ACS}_{M,ppi}, K^{ACS}_{M,amp}, K_{eq}, h, N = 3]$
Rank 3, $K^{MALS}_{M,accoa}$	MALS: $glx_c + accoa_c \rightleftharpoons mal_{L_c} + coa_c$	$v = \frac{V_{max}\left(1-\frac{1}{K_{eq}}\prod_{i=1}^{N}\left(\frac{[P_i]}{[S_i]}\right)\right)\prod_{i=1}^{N}\left(\frac{[S_i]}{K_{M,S_i}}\right)\left(\frac{[S_i]}{K_{M,S_i}}+\frac{[P_i]}{K_{M,P_i}}\right)^{h-1}}{\prod_{i=1}^{N}\left(1+\left(\frac{[S_i]}{K_{M,S_i}}+\frac{[P_i]}{K_{M,P_i}}\right)^{h}\right)}$ Parameters: $[V_{max}, K^{MALS}_{M,glx}, K^{MALS}_{M,accoa}, K^{MALS}_{M,mal_{L_c}}, K^{MALS}_{M,coa},, K_{eq}, h, N = 2]$

Rank 4, $K_{M,accoa}^{PFL}$	PFL: coa_c+pyr_c $\rightleftharpoons for_c + accoa_c$	$$v = \frac{V_{max}\left(1 - \frac{1}{K_{eq}}\prod_{i=1}^{N}\left(\frac{[P_i]}{[S_i]}\right)\right)\prod_{i=1}^{N}\left(\frac{[S_i]}{K_{M,S_i}}\right)\left(\frac{[S_i]}{K_{M,S_i}} + \frac{[P_i]}{K_{M,P_i}}\right)^{h-1}}{\prod_{i=1}^{N}\left(1 + \left(\frac{[S_i]}{K_{M,S_i}} + \frac{[P_i]}{K_{M,P_i}}\right)^{h}\right)}$$ Parameters: $[V_{max}, K_{M,coa}^{PFL}, K_{M,pyr}^{PFL}, K_{M,for_c}^{PFL}, K_{M,accoa}^{PFL}, K_{eq}, h, N = 2]$
Rank 5, $K_{M,q8}^{SUCDi}$	SUCDi: $q8_c$+$succ_c$ $\rightleftharpoons fum_c + q8h2_c$	$$v = \frac{V_{max}\left(1 - \frac{1}{K_{eq}}\prod_{i=1}^{N}\left(\frac{[P_i]}{[S_i]}\right)\right)\prod_{i=1}^{N}\left(\frac{[S_i]}{K_{M,S_i}}\right)\left(\frac{[S_i]}{K_{M,S_i}} + \frac{[P_i]}{K_{M,P_i}}\right)^{h-1}}{\prod_{i=1}^{N}\left(1 + \left(\frac{[S_i]}{K_{M,S_i}} + \frac{[P_i]}{K_{M,P_i}}\right)^{h}\right)}$$ Parameters: $[V_{max}, K_{M,q8}^{SUCDi}, K_{M,succ}^{SUCDi}, K_{M,fum}^{SUCDi}, K_{M,q8h2}^{SUCDi}, K_{eq}, h, N = 2]$
Rank 6, $K_{M,icit}^{ICL}$	ICL: $icit_c$ $\rightleftharpoons succ_c + glx_c$	$$v = \frac{V_{max}\prod_{i=1}^{M}\left(\frac{[S_i]}{K_{M,S_i}}\right)\left(1 - \frac{1}{K_{eq}}\frac{\prod_{j=1}^{N}[P_j]}{\prod_{i=1}^{M}[S_i]}\right)}{\prod_{i=1}^{M}\sum_{m=0}^{\alpha_i}\left(\frac{[S_i]}{K_{M,S_i}}\right)^{m} + \prod_{j=1}^{N}\sum_{m=0}^{\beta_j}\left(\frac{[P_j]}{K_{M,P_j}}\right)^{m} - 1}$$ Parameters: $[V_{max}, K_{M,icit}^{ICL}, K_{M,succ}^{ICL}, K_{M,q8h2}^{ICL}, K_{eq}, N = 2, M = 1, \alpha_1 = 1, \beta_1 = -1, \beta_2 = -1]$
Rank 7, $K_{M,co2}^{CO2tpp}$	CO2tpp: $co2_e$ $\rightleftharpoons co2_c$	$$v = \frac{V_{max}\left(1 - \frac{1}{K_{eq}}\prod_{i=1}^{N}\left(\frac{[P_i]}{[S_i]}\right)\right)\prod_{i=1}^{N}\left(\frac{[S_i]}{K_{M,S_i}}\right)\left(\frac{[S_i]}{K_{M,S_i}} + \frac{[P_i]}{K_{M,P_i}}\right)^{h-1}}{\prod_{i=1}^{N}\left(1 + \left(\frac{[S_i]}{K_{M,S_i}} + \frac{[P_i]}{K_{M,P_i}}\right)^{h}\right)}$$ Parameters: $[V_{max}, K_{M,co2_c}^{CO2tpp}, K_{M,co2_e}^{CO2tpp}\ K_{eq}, h, N = 1]$

Supplementary Table 1: The reaction and the rate law formalisms of the top 7 kinetic parameters from Fig. 3a. Here S_i and P_i represents the ith substrate and product of a reaction, K_{eq} represents the equilibrium constant of the reaction and h is the Hill coefficient of the generalized Michaelis-Menten and Convenience kinetics.

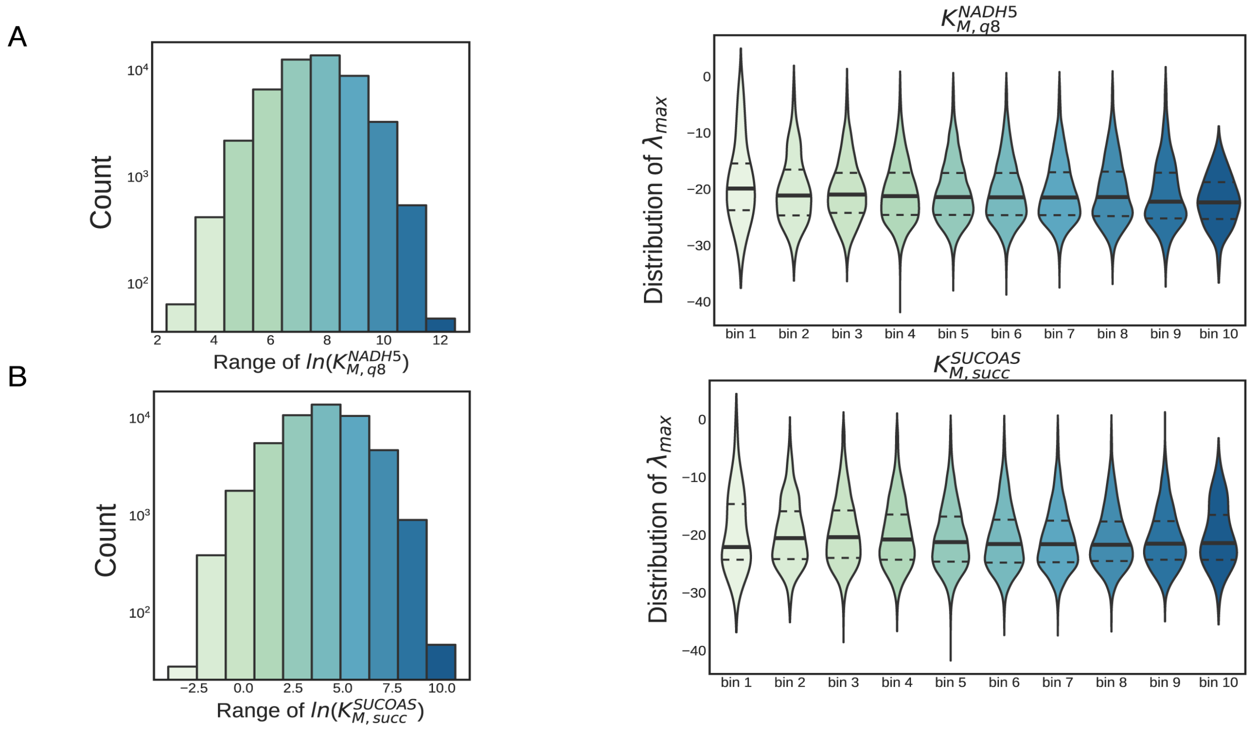

Supplementary Figure 7: 50,000 REKINDLE generated models were divided into 10 sub-populations (indicated by the different shades of the bins) based on their respective values of A) $K_{M,q8}^{NADH5}$ B) $K_{M,suc}^{SUCOAS}$ (left). and (Right) the maximum eigenvalue distributions of the Jacobian for the subpopulations. The thick black line indicates the mean of the distribution and the dashed lines indicates quartiles. These are the lowest ranked parameters in Fig 3a (main manuscript) and consequently show negligible effect on the dynamics compared to high ranked parameters.

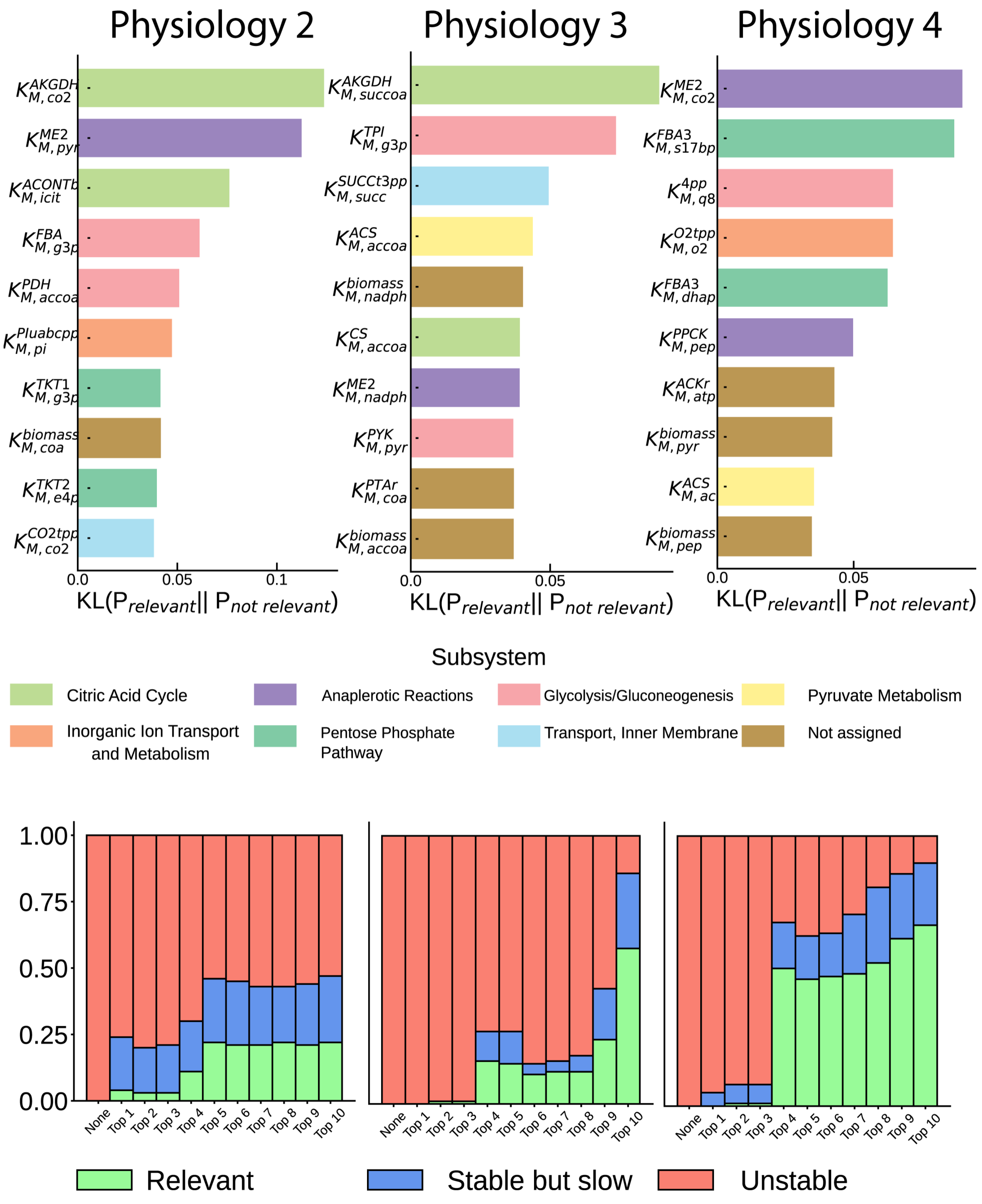

Supplementary Figure 8: Top: KL divergence score between distributions of the relevant and non-relevant class of generated kinetic parameters for Physiology 2 (left), Physiology 3 (middle), Physiology 4 (right) and the respective metabolic subsystems thy belong to. Bottom: We begin with 100 locally unstable models (orange bar, left) for each

physiology and gradually constrain the ranked parameters in the top panel in a cumulative manner, based on their ranks (right). Constraining the parameters stabilizes (blue) as well as rescues (green) the unstable models.

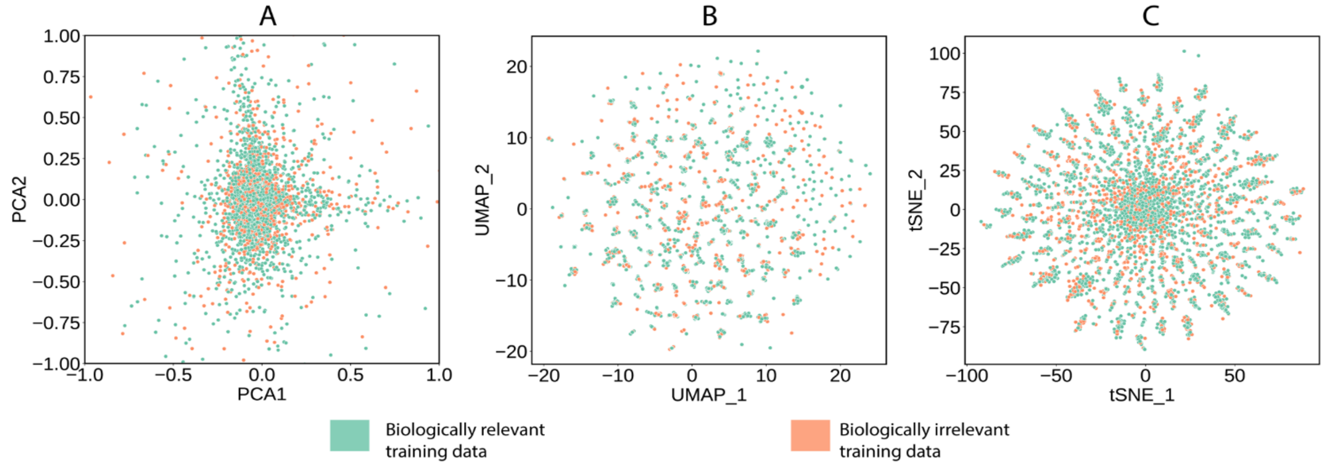

Supplementary Figure 9: The kinetic parameter space of the training dataset for Physiology 1 was visualized using different unsupervised dimension reduction tools (a) PCA (b) UMAP (c) tSNE. We observe that these tools are not able to recover a proper boundary between the relevant and non-relevant kinetic parameter sets. We observed similar results for all the 4 different physiologies.

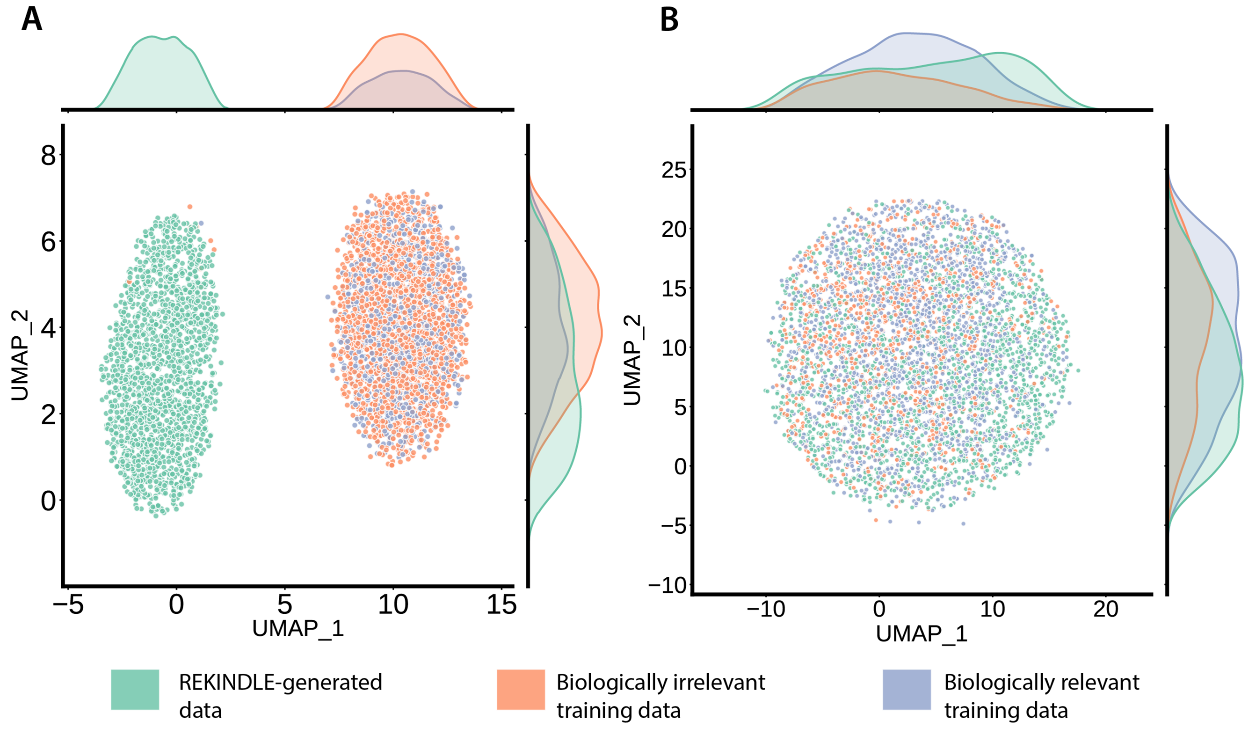

Supplementary Figure 10: The kinetic parameter space was visualized using UMAP (a) with all the parameters (b) Only the top 5 parameters in Fig 3a. We observe that in the (a) the REKINDLE generated data (green) and the training set (orange and blue) are completely disjoint and in (b) they share the same space. This indicates that the GANs in REKINDLE are able to distinguish between significant parameters and non-significant parameters and learn them accordingly.

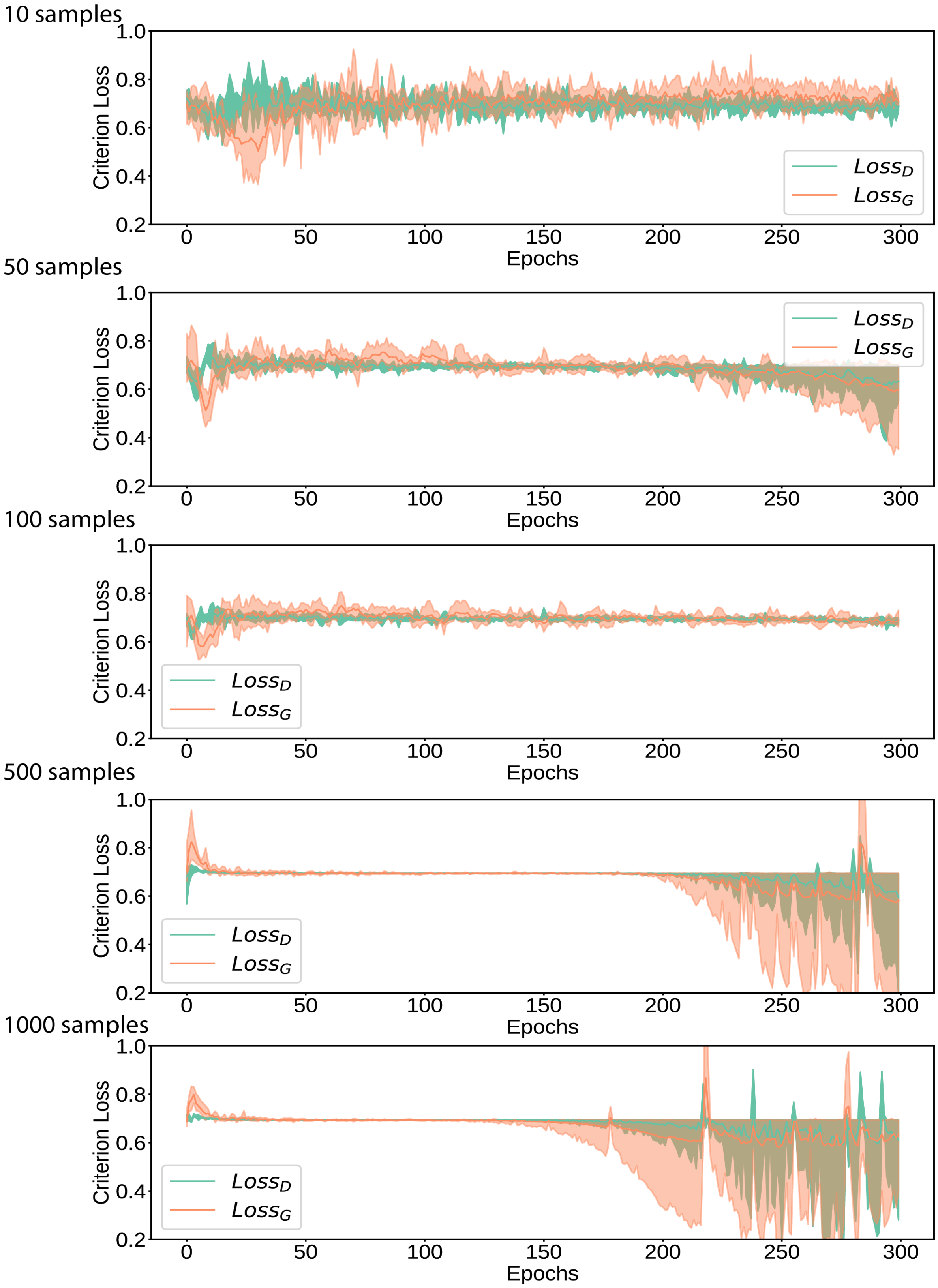

Supplementary Figure 11a: The discriminator loss (green line) and generator loss (orange line) for the case of transfer learning from Physiology 4 to Physiology 1, using 10 (top panel), 50, 100, 500 and 1000 (bottom panel) samples from physiology 1, for 5 statistical repeats. The losses show similar trends for all 11 other cases of transfer learning.

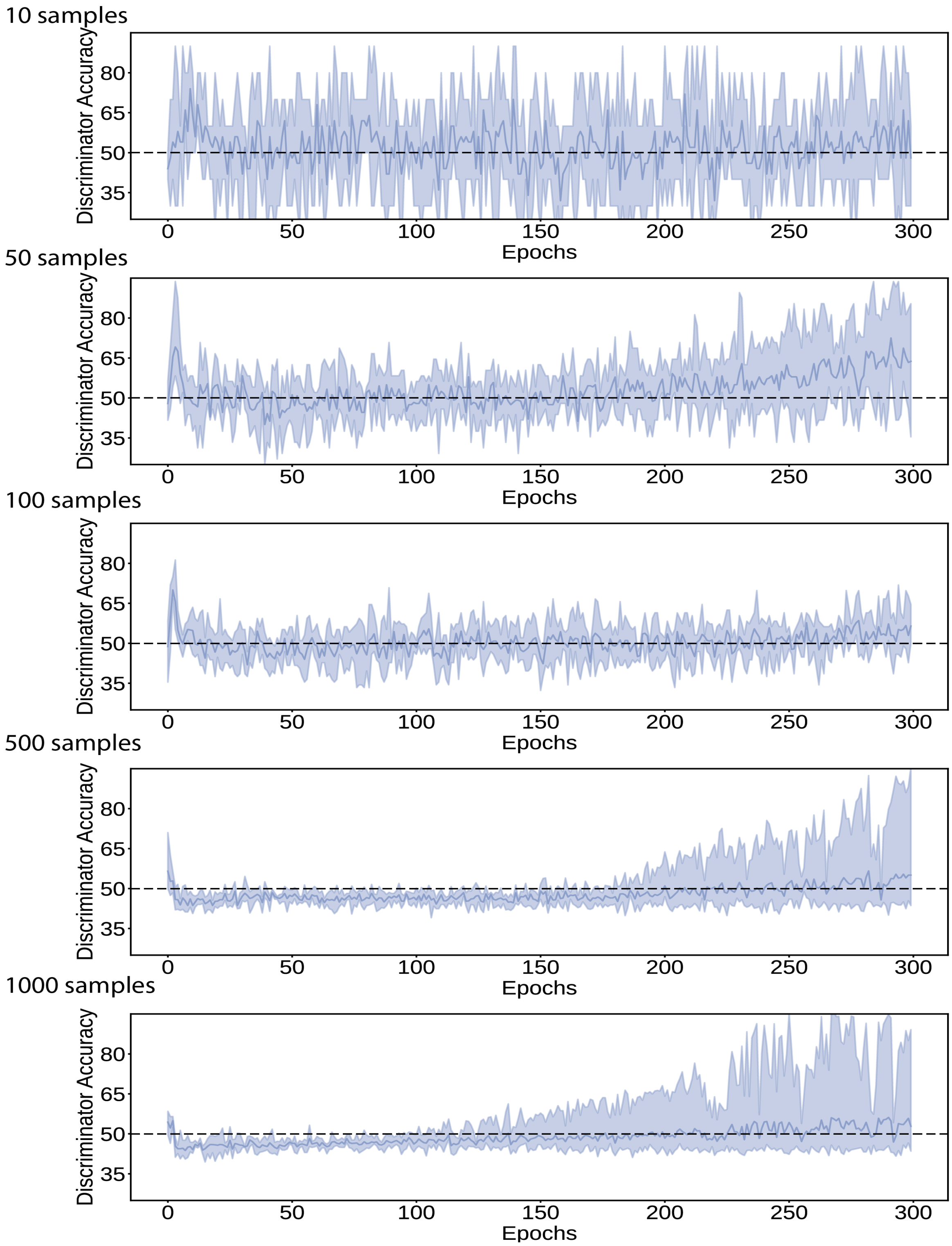

Supplementary Figure 11b: The discriminator accuracy for the case of transfer learning from Physiology 4 to Physiology 1, using 10 (top panel), 50, 100, 500 and 1000 (bottom panel) samples from physiology 1, for 5 statistical repeats. The accuracies show similar trends for all 11 other cases of transfer learning. It can be seen that training beyond 150 epochs leads to discriminator overpowering the generator for the cases with 500 and 1000 samples.

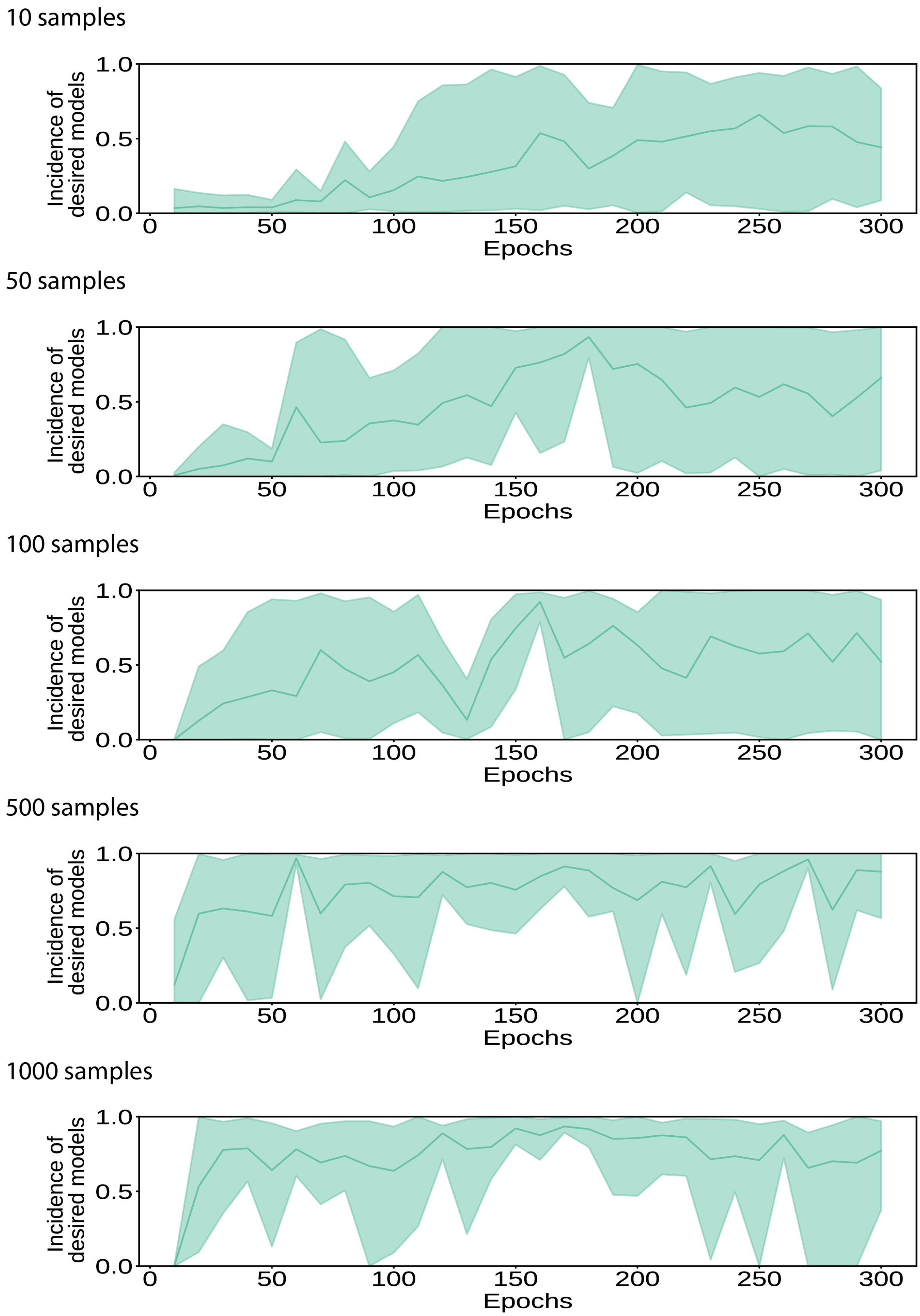

Supplementary Figure 11c: The incidence of biologically relevant models for the case of transfer learning from Physiology 4 to Physiology 1, using 10 (top panel), 50, 100, 500 and 1000 (bottom panel) samples from physiology 1, for 5 statistical repeats. The incidences show similar trends for all 11 other cases of transfer learning.

Supplementary Note 1: Testing classification performance of different machine learning algorithms on kinetic parameter sets

The choice of the proper machine learning algorithm was crucial in accomplishing our task of conditional generation of a specific category of training data. For conditional generation, successful implicit classification (between different categories of training data) is a necessary condition. As GANs accomplish both these tasks, it was our method of choice. Variational Autoencoders (VAEs)[2], another class of generative neural networks with similar complexity as GANs, would also be an attractive option but we opted for GANs because of its ability to extrapolate data[3].
Nevertheless, we did a comparative study of the classification accuracy (the ability to correctly classify biologically relevant and non-relevant kinetic parameter sets for the 1st physiology) of four commonly used algorithms (I) Logistic Regression (II) Support Vector Machines (III) XGBoost Classifier (IV) Random Forest Classifier. The classification performance is summarized in Supplementary Figure 12.

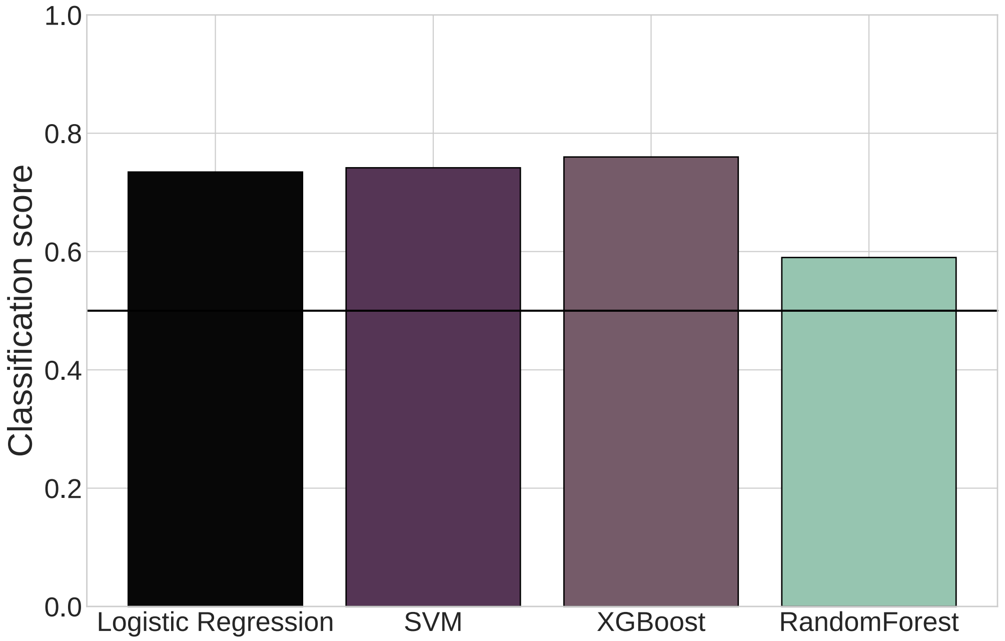

Supplementary Figure 12: Classification accuracies of standard machine learning models between biologically relevant and irrelevant kinetic parameter sets for physiology 1. The black horizontal line indicates 50% classification accuracy.

None of these methods had a classification accuracy superior to 75 % ((I) Logistic Regression: 73.46 % (II) Support Vector Machines 74.28% (III) XGBoost Classifier 75.71% (IV) Random Forest Classifier 59%), indicating that more complex nonlinear function approximators like deep neural networks are more suited for this task. Additionally, these methods did not provide reliable information about important features that one could use for the downstream task of conditional generation. We pruned the important features indicated by the algorithms mentioned above, and this pruning did not lead to an increase in performance. This indicates that the feature importance results are unreliable due to low classification accuracy and hence will lead to inconsistent conclusions if used for the generation task. Finally, even if these simpler algorithms had good classification accuracy, it would be difficult for some of these to integrate the information from the classifier

into the kinetic modeling frameworks. This study was implemented in Python using the scikit-learn library (https://scikit-learn.org/stable/) and is available in the Github repository of REKINDLE (https://github.com/EPFL-LCSB/rekindle) named `algorithm_classification_test.ipynb`.

Supplementary Note 2: Advantages of REKINDLE over traditional sampling-based kinetic modeling methods.

REKINDLE offer the following benefits over traditional sampling-based kinetic modeling methods:

I. **Ease of extrapolation (transfer learning) to other physiological or steady-state conditions:** Even when sampling-based workflows like ORACLE have a good incidence of kinetic models with desired dynamics for one specific steady-state condition, it does not guarantee similarly good incidences for other conditions. In contrast, REKINDLE can consistently extrapolate and generate models with desired dynamics over multiple steady-state and physiological conditions using transfer learning.

II. **'Off the shelf' usage:** The state of a trained REKINDLE generator for a given physiological condition can be saved and then reused indefinitely to generate models with desired dynamics. Implicit knowledge about the system, such as the metabolic network structure and non-linearities of the enzyme mechanisms, are inherently captured and stored in the state of the neural network. This allows for the creation of openly accessible repositories of neural networks specific for various physiological conditions/ specific strains & organisms that the community can use directly.

III. **Generation Time:** Generation with neural networks is orders of magnitude faster than sampling based kinetic networks.

IV. **Customizable Stopping Criteria:** The duration and quality of the training can be customized by defining criteria for specific properties of the generated data (here, one can define desired thresholds for metrics like KL divergence, diversity of generated dynamic modes, etc.), to choose the best generator for downstream studies automatically.

Supplementary Note 3: Checking for overfitting in GANs

We have reported in the manuscript the distribution of the eigenvalues corresponding to the slowest dynamics for the models trained from scratch and the transfer learning trained models (Fig 4c, lower panel). The distributions show that parameter sets generated by transfer learning are as diverse as those obtained by training from scratch, and none of the generators obtained from transfer learning has a mode collapse. These indicate that there is no overfitting in the generator.

Next, to address overfitting in the discriminator, we cross-checked our results with literature from the field. Overfitting in the discriminator is indicated by an increase in the statistical distance between generated and training data[4] and an increase in discriminator accuracy, as an overfitted discriminator tends to reject generated data points that are slightly different from the training data, thus biasing the generator into a mode collapse or a failure in the training procedure. A mode collapse results when the generator always generates

the same model and the discriminator accepts it. This can be verified by inspecting at the diversity of the generated models. We observe that the generated models have a high diversity in terms of the dynamic modes of the Jacobian (Figure 2a, Figure 4c bottom panel, Supplementary Figure 3a, 3b, 3c). Moreover, the distributions of the individual kinetic parameters generated are also broad (Figure 3a), indicating there was not a mode collapse.

Moreover, we see a decreasing trend in the KL divergence score between the training data and generated data (Fig 2a, Supplementary Figure 3a, 4a, and 5a, Supplementary Note 3) and convergence of the discriminator accuracy to near 50 % (Supplementary Figure 2), indicating that the major indicators of discriminator overfitting are absent. We acknowledge that a decreasing statistical distance between two distributions does not guarantee diversity of populations (e.g., the distance between two very similar but narrow distributions is also zero/close to zero). However, since we measure the KL divergence with respect to the training data, which is diverse enough for our purposes, a decreasing KL divergence indicates that generated data is also approaching the same diversity.

Similarly, in the case of transfer learning in REKINDLE-TL, we have plotted the discriminator accuracies for one of the 12 cases (Supplementary Figure 11b). As mentioned in the manuscript, the other eleven cases followed the same trend. For the cases with 500 and 1000 training data samples, we see overfitting in the later stages of training (after 200 and 150 epochs, respectively) indicated by the increasing discriminator accuracy. This is also accompanied by more significant fluctuations in the criterion losses of the discriminator and generator (Supplementary Figure 11a). However, we also observe that our desired incidence of relevant models converges to near 100 %, well before the overfitting commences (around 50 epochs). Thus, we only used results from the earlier phase of the training to avoid using generated models from a pathological stage of the training process.

Supplementary Note 4: Validating statistical similarity of generated data against test dataset and randomized dataset.

In Figure 2a, Supplementary Figure 3a, 4a, 5a, we report the Kullback-Leibler divergence of the generated dataset from the training dataset, confirming that the generated data becomes statistically similar to the training data as training progresses. For additional validation, we measured the KL divergence of the generated data with the test dataset as well (Methods). We expected to see similar trends in the KL divergence as the training and test sets are statistically identical. This was confirmed by plotting the divergences for both the datasets as shown in Supplementary Figure 13.

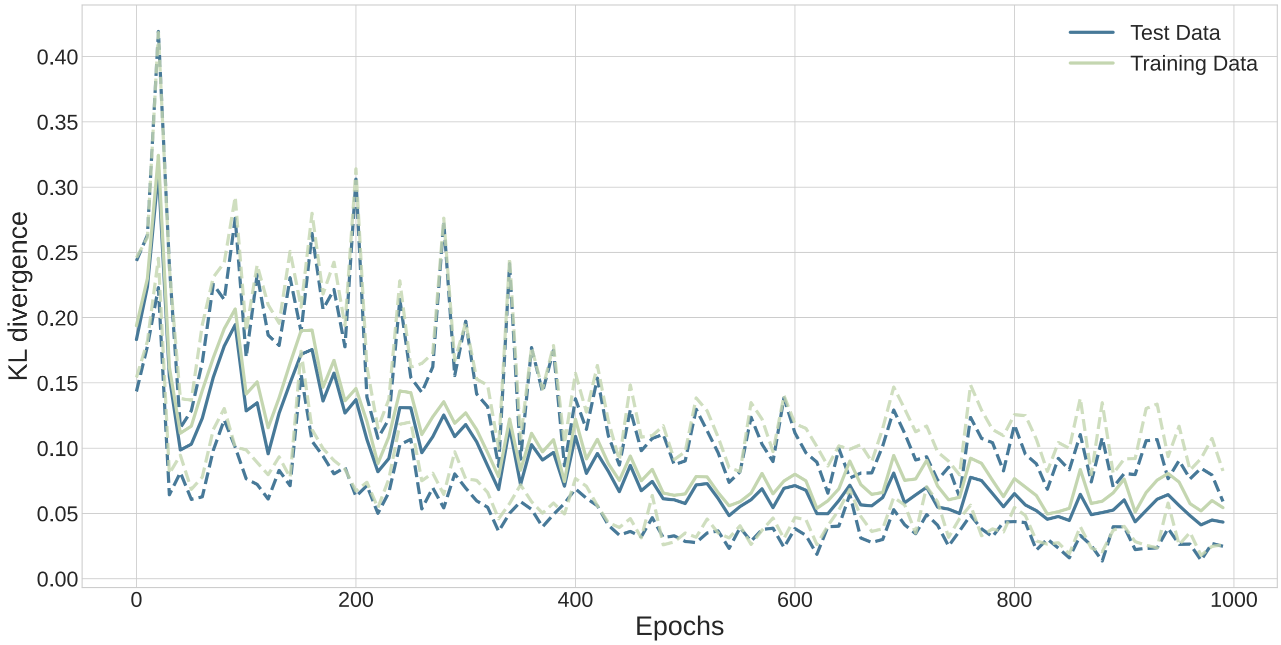

Supplementary Figure 13: Kullback Leibler divergence between the REKINDLE generated data and (i) training data (solid light green) (ii) test data (solid teal line) for Physiology 1 for 5 statistical repeats. The corresponding dashed lines indicate the minimum and maximum KL divergences observed over 5 repeats.

We observe that the KL divergences show a decreasing trend in both cases, indicating that the GANs did not overfit the training data. For further validation, we generated a random control dataset of 5000 kinetic parameter sets by uniformly sampling every individual kinetic parameter independently, within their respective ranges in the ORACLE dataset. We then calculated the KL divergence of the generated data and the control dataset and compared it with the training and test datasets. The results are summarized in Supplementary Figure 14. We observe that the KL divergence between the REKINDLE generated dataset and the control dataset does not show any decreasing trend unlike as in the case of training and test datasets. This implies that the decreasing trend in the latter cases is not a random artefact but a result of successful learning of the biologically relevant parameter space by the GANs during training.

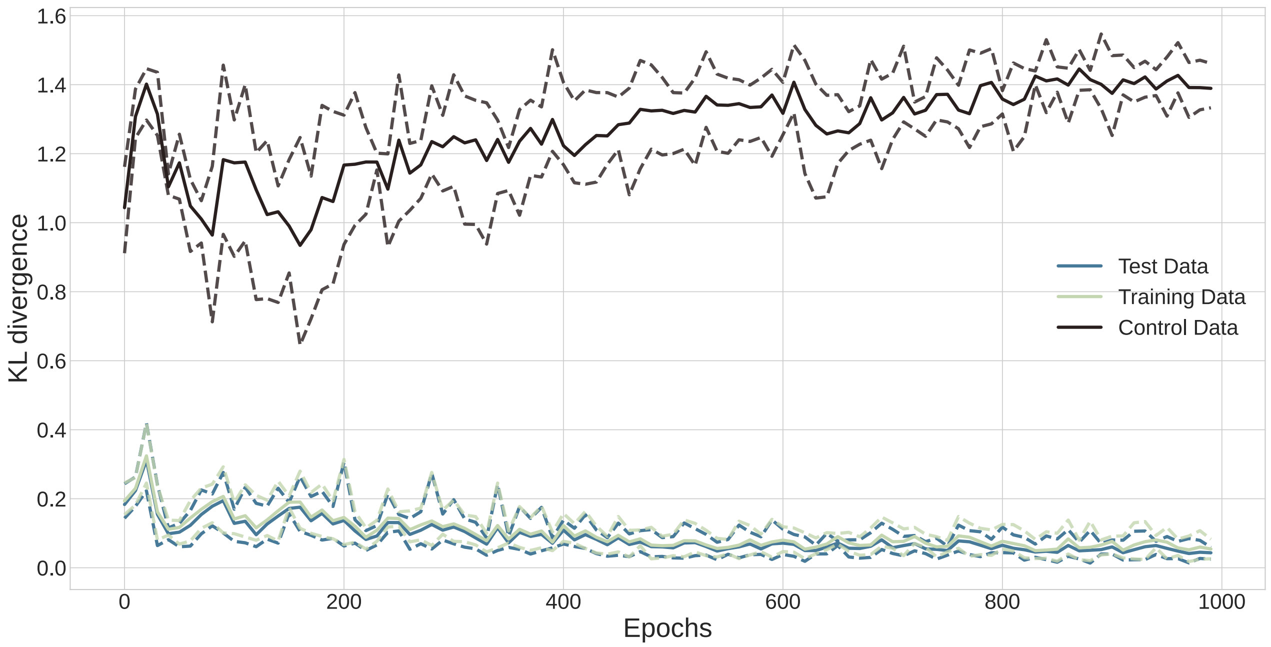

Supplementary Figure 14: Kullback Leibler divergence between the REKINDLE generated data and (i) training data (solid light green) (ii) test data (solid teal line) (iii) control data (black-brown line) for Physiology 1 for 5 statistical repeats. The corresponding dashed lines indicate the minimum and maximum KL divergences observed over 5 repeats.

Supplementary Note 5: Generating biologically non-relevant models with REKINDLE.

Conditional Generative Adversarial Networks (cGANs) can be used to generate new data from a specific labelled class of the training data (biologically-relevant, biologically-nonrelevant). In REKINDLE, we use cGANs to conditionally generate biologically relevant kinetic parameter sets using the biologically-relevant class label in the generator seed. While we are not interested in biologically-nonrelevant models because they do not represent any physiologically observed metabolic characteristics, for the sake of completion, we investigated can we generate non-relevant models using the corresponding class label in REKINDLE. We selected the generators with the highest incidence of biologically relevant models (Supplementary Table 2) and generated 1000 non-relevant models for each metabolic physiology by changing the class label in the generator seed. We then tested these models for non-relevance by calculating the eigenvalues of their Jacobian matrix (Methods). The results are summarized below (Supplementary Table 2).

We observed that the incidence of non-relevant models is low compared to relevant models. This suggests that the cGANs did not learn the non-relevant space of the kinetic parameter space as well as it did for the relevant space. To investigate this space further, we discretized the kinetic parameter space of the training data into 16 subspaces based on the maximum eigenvalue of the Jacobian near the boundary line of our imposed classes i.e., $Re(\lambda_{max}) = -9,$ for all 4 physiologies (X axis, Supplementary Figure 15 a-d). For each of these 16 subspaces, we quantified the difference between the distributions of individual kinetic parameters that belong to a particular subspace and those subspaces that have larger eigenvalues, again using the KL divergence score. We observed that only locally stable subspaces ($Re(\lambda_{max}) < 0$) contained kinetic parameter sets that had significant differences in the distributions of certain kinetic parameters whereas non-relevant and especially locally unstable models ($Re(\lambda_{max}) > 0$) did not (Supplementary Figure 15 a-d, here

the KL divergence scores are normalized for each subspace). This suggests that there are no determining characteristic parameters that make a certain kinetic parameter set locally unstable, unlike relevant parameter sets.

Supplementary Table 2: Incidence of biologically relevant and non-relevant models generated with REKINDLE for four physiologies.

	Physiology1	Physiology 2	Physiology 3	Physiology 4
Directionality of Reactions	$\underset{\rightarrow}{TALA}\ \underset{\rightarrow}{ICL}$	$\underset{\rightarrow}{TALA}\ \underset{\leftarrow}{ICL}$	$\underset{\leftarrow}{TALA}\ \underset{\rightarrow}{ICL}$	$\underset{\leftarrow}{TALA}\ \underset{\leftarrow}{ICL}$
Relevant Models	97.7%	97.3%	99.3%	100.0%
Non-relevant Models	18.5%	9.8%	54.5%	16%
(stable but slow, unstable)	(6.1%, 12.4%)	(3%, 6.8%)	(30.2%, 24.3%)	(11.1%, 4.9%)

Moreover, we also plotted the same heatmap as Supplementary Figure 15 but this time the KL divergence scores were normalized over the entire space and not for each subspace, to visualize where the strongest information was present. We observed that the strongest signals in the KL divergence scores of the relevant models appear further away from the eigenvalue partition line ($Re(\lambda_{max}) = -9$) for 3 of the 4 physiologies ($Re(\lambda_{max}) \sim -20$ for physiology 1, $Re(\lambda_{max}) \sim -25$ for physiology 3 and $Re(\lambda_{max}) \sim -25$ for physiology 4 (Supplementary Figure 16 a-d). We hypothesize that the GANs used in REKINDLE is able to capture this bias and thus generates kinetic parameter sets that are further away from the eigenvalue class partition line compared to the training data (Figure 2e, Supplementary Figure 3a-c). Exceptionally, the strongest signal for physiology 2 occurs around $Re(\lambda_{max}) = 10$ (Supplementary Figure 15b, Supplementary Figure 16b).

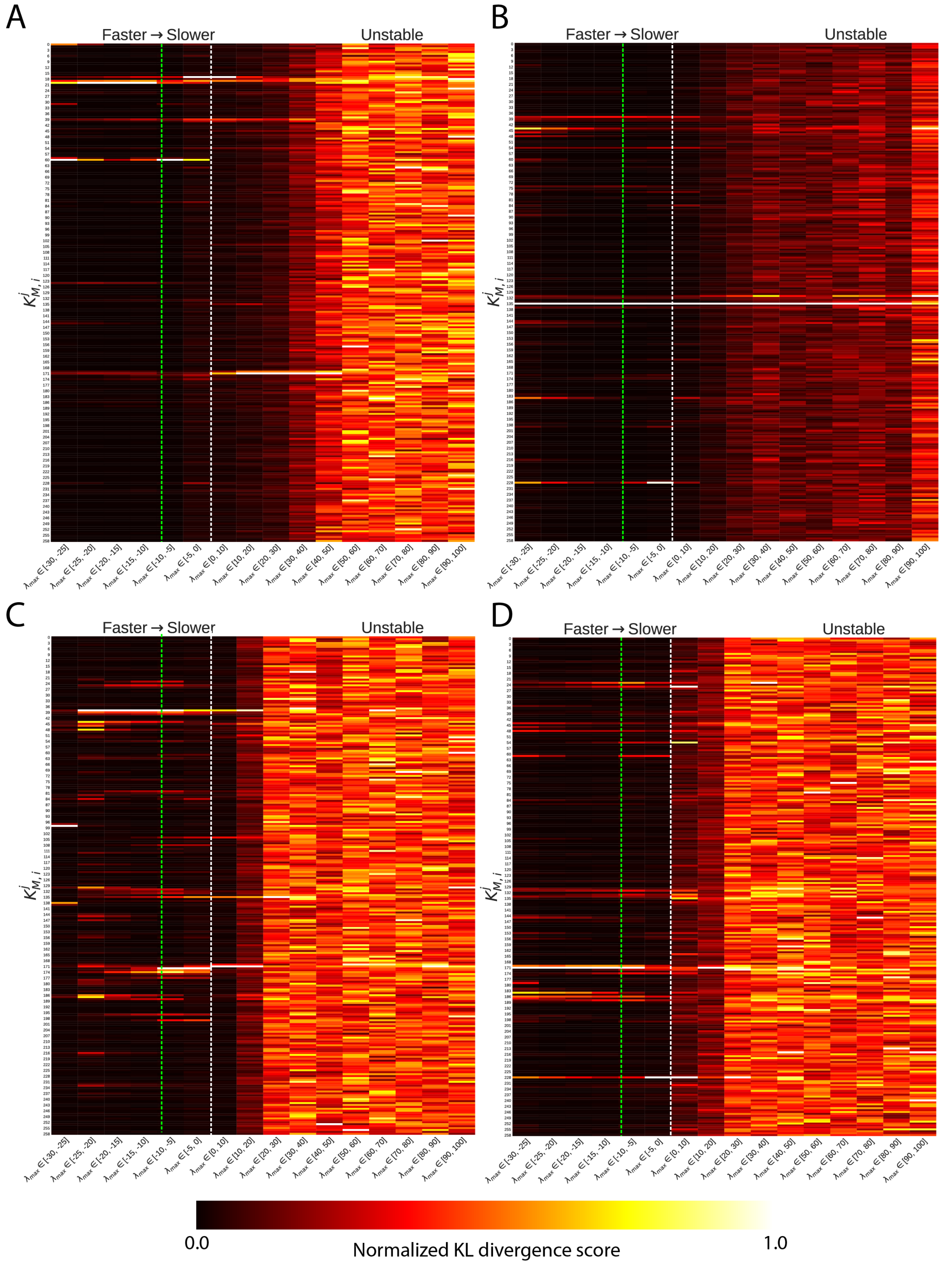

Supplementary Figure 15: Differences in distributions of individual kinetic parameters (y-axis) using KL divergence between 16 discretized sub-populations based on eigenvalue space (x-axis) near the class boundary i.e., $Re(\lambda_{max}) = -9$ (green dashed line) for (a) Physiology 1 (b) Physiology 2 (c) Physiology 3 (d) Physiology 4. The white dashed line represents $Re(\lambda_{max}) = 0$. Here the KL divergence for each sub-population is normalized between 0 and 1.

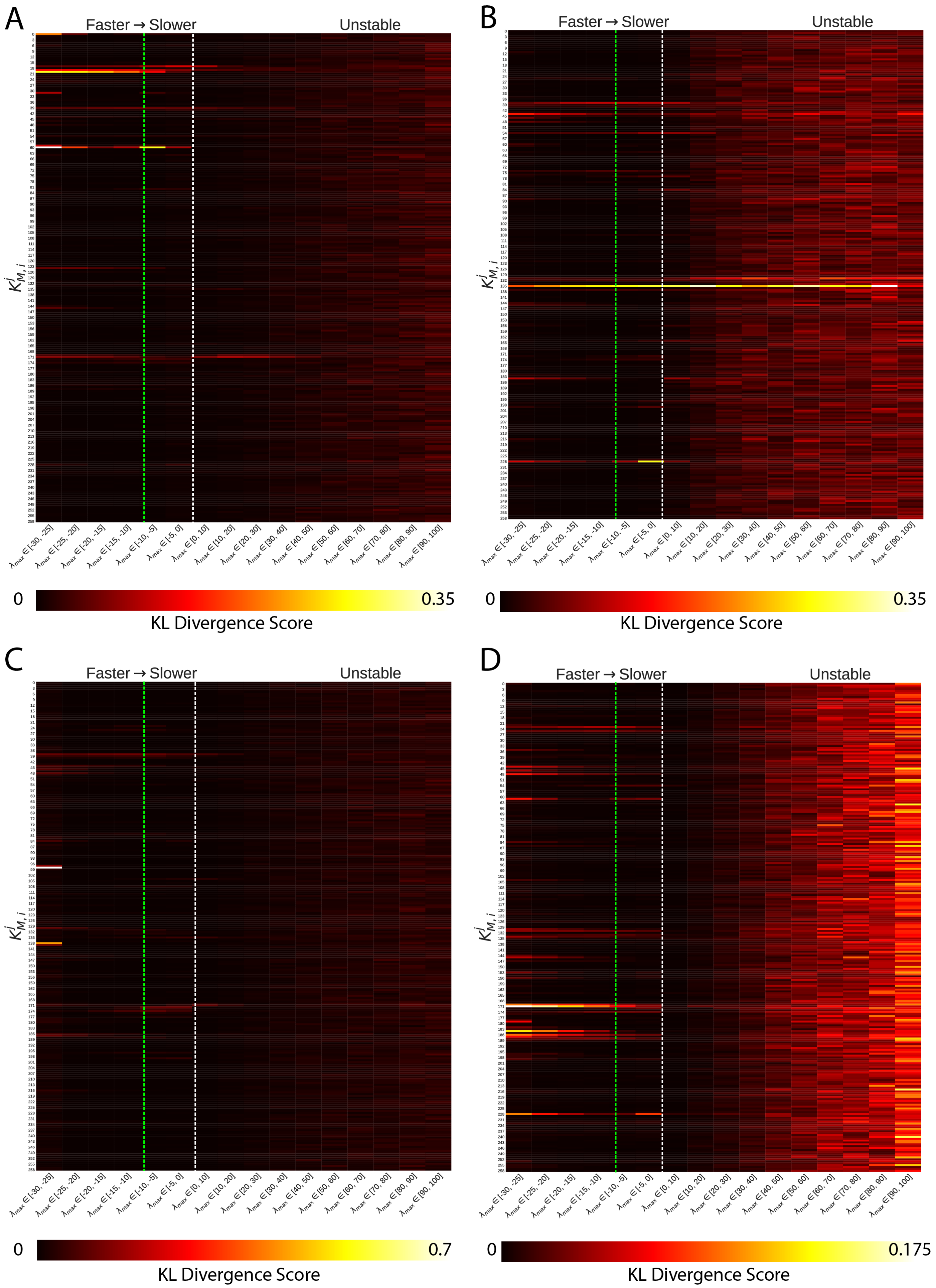

Supplementary Figure 16: Differences in distributions of individual kinetic parameters (y-axis) using KL divergence between 16 discretized sub-populations based on eigenvalue space (x-axis) near the class boundary i.e., $Re(\lambda_{max}) = -9$

(green dashed line) for (a) Physiology 1 (b) Physiology 2 (c) Physiology 3 (d) Physiology 4. The white dashed line represents $Re(\lambda_{max}) = 0$.

Supplementary Note 6: Interpretability of REKINDLE results

REKINDLE captures the complex mapping between the kinetic parameters and the biologically relevant dynamics space, and interpreting its outputs offers insights into the relevant kinetic parameters that ensure imposed physio-biological conditions.

The comparison of KL divergence scores of biologically relevant and irrelevant kinetic models (2000 kinetic models from each category) for Physiology 1 revealed that just a few parameters had a significant difference in the distributions between the two populations (Supplementary Figure 5a, b). This suggests that the values of these parameters are indeed affecting the biological relevance of the generated models.

We took a random population of 100 locally unstable models to test this hypothesis. Within this population, we constrained the highest-ranked parameter, $K_{M,atp}^{ACS}$ (Fig 3a, Supplementary Figure 6, Supplementary Table 1), within its top 10 percentile values in the direction that favors relevancy, i.e., toward higher parameter values (Supplementary Figure 17, Figure 3), and observed the dynamics of the resulting kinetic models. Remarkably, 29 of the resulting models became biologically relevant, meaning that this parameter strongly affects this property. We repeated this procedure and constrained the top 2 parameters, the top 3 parameters, and so on until we constrained all ten of them. The constraints on the parameters resulted in a bigger portion of models becoming biologically relevant. For example, when we constrained the top 7 parameters, we obtained ~60% of models with biologically relevant dynamics and over 70% of locally stable models. Adding further constraints beyond the top 7 parameters did not significantly affect the dynamics, indicating that these parameters are sloppy.

We repeated this study for the other three physiologies (Supplementary Figure 8). Similar to Physiology 1, in Physiologies 2 – 4, the parameter group with the highest KL divergence scores had the highest impact on the biological relevance. While a high KL divergence score indicated that a kinetic parameter was affecting the biological relevance, the parameter ranking on this score did not always coincide with the most efficient conversion of biologically irrelevant models to relevancy. For example, we observed for Physiology 3 that biologically irrelevant models became relevant only after constraining the first five parameters (Supplementary Figure 8), suggesting that the 5th ranked parameter was the most significant for biological relevancy.

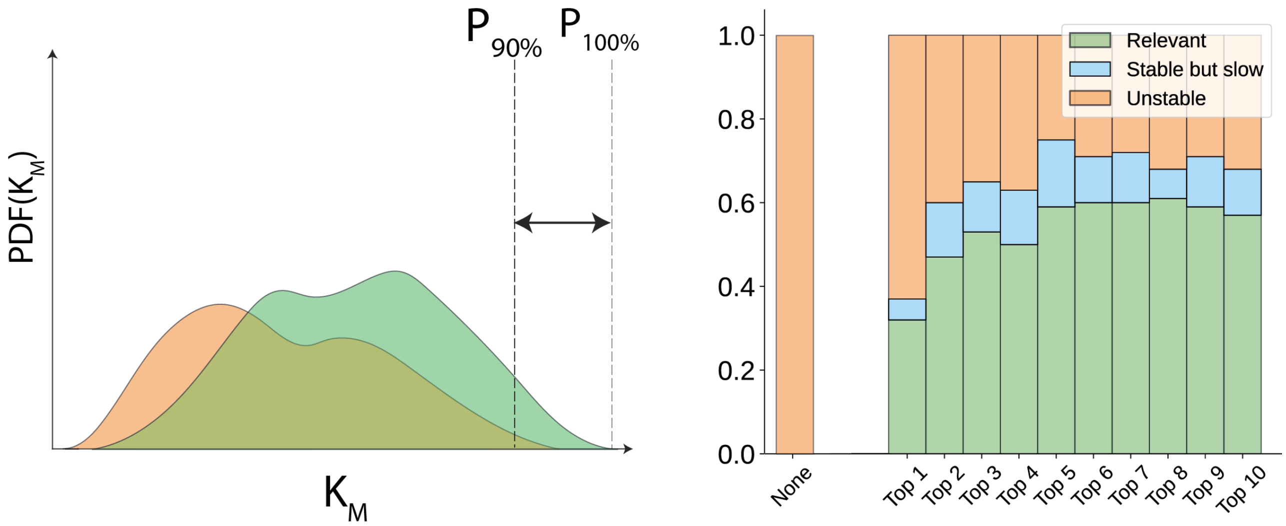

Supplementary Figure 17: (Left) The parameters in Fig 3a (main manuscript) are constrained within 90-th percentile and 100-th percentile (dashed vertical lines) of the relevant class (green) in the direction that favors biological relevancy. **(Right)** The fraction of locally unstable, stable and relevant models as Fig 3a result of constraining the parameters as explained in the left panel. We begin with 100 locally unstable models (orange bar, left) and gradually constrain the parameters in a cumulative manner, based on their ranks (right). Constraining the parameters stabilizes (blue) as well as rescues (green) the unstable models. We notice that there is a negligible change in dynamics after the top 7 parameters have been constrained.

These results suggest that GANs trained in REKINDLE are capable of implicit model reduction and distinguishing between features (kinetic parameters) that directly affect desired properties from features that do not. To confirm this, we performed unsupervised dimension reduction with UMAP[41] on the REKINDLE generated set and the training set for Physiology 1, first using all the parameters and then using only the top 5 parameters from Fig. 3a (Supplementary Figure 10a, b). In the former case, we observed that the generated and the training set are disjoint, while in the latter case, both sets share the same space.

Supplementary Note 7: Differences in kinetic parameter distributions between different physiologies.

The differences in individual parameter distributions between the different physiologies is a relevant metric, especially in transfer learning. To examine this, we looked at the statistical difference between the distributions of individual kinetic parameters between different physiologies, quantified using the KL divergence. The results are summarized in Supplementary Figure 18.

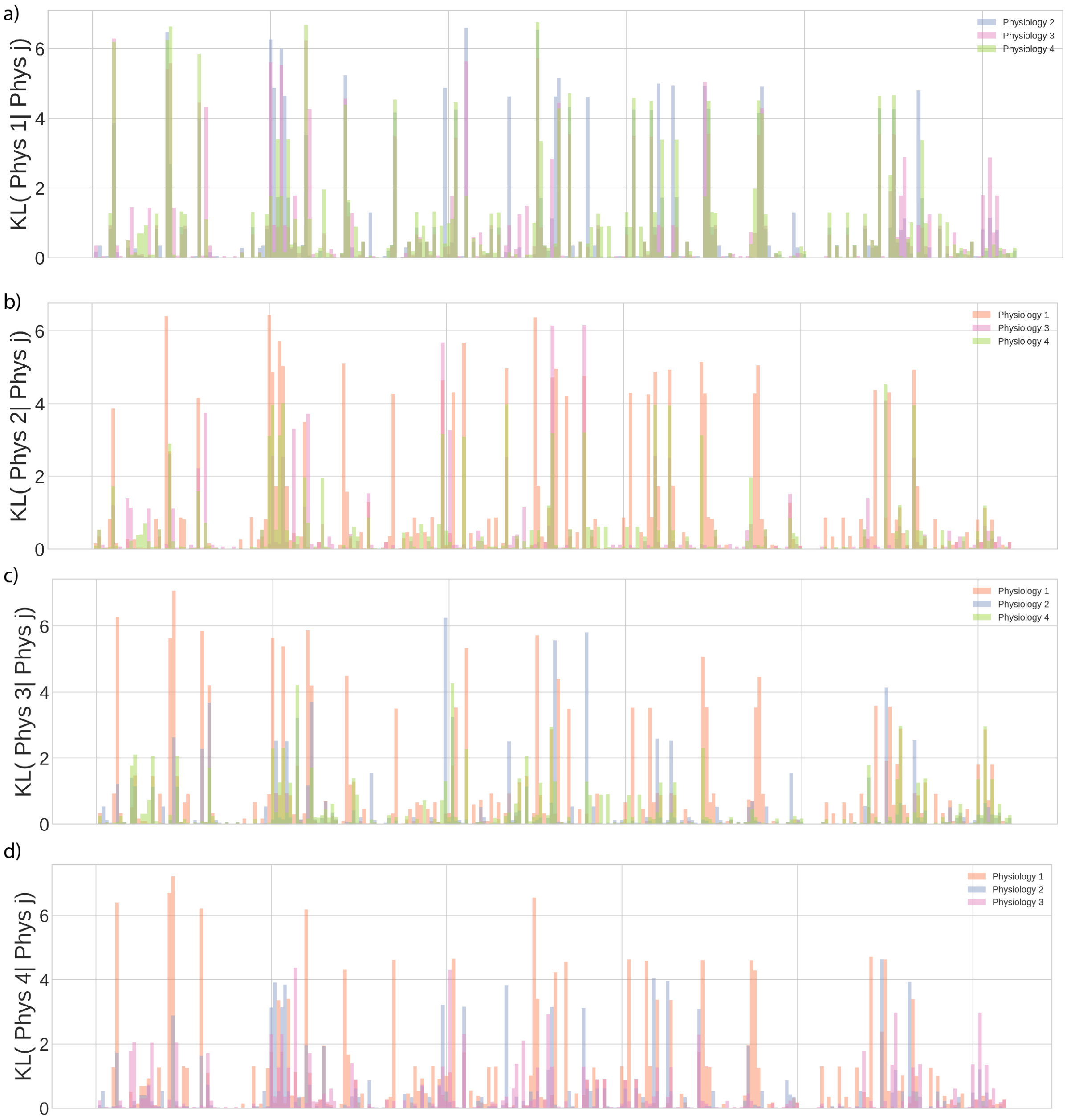

Supplementary Figure 18: The KL divergences between distributions of 259 individual kinetic parameters, parameterized by ORACLE, among the 4 physiologies used.

As expected, we observe that some of the parameters are completely sloppy (very similar distributions between physiologies). However, many of them are affected by the changes in the directionality of reactions in the metabolic network. The transfer learning manages to capture these differences and generate relevant models accordingly for all the physiologies. Additional indicator of the differences between the parameter sets of different physiologies are the top ranked parameters based on the difference in distributions of relevant and non-relevant parameter sets (Figure 3 and Supplementary Figure 17).

Supplementary Note 8: Extrapolation beyond test and training datasets.

In Supplementary Note 4, we show that the generated dataset well generalizes the entire ORACLE dataset from which the training and test data were sourced. But this is not indicative of any incidence of data being generated outside the domain of the original dataset. To examine any evidence of REKINDLE generalizing beyond the test and training set, we looked at the eigenvalue distributions of the generated datasets during training and compared their width of distributions to the test and training dataset (Supplementary Figure 19). We observe that in some instances, the generated models go well beyond the ORACLE dataset. However, the generators with the capacity to produce models beyond the test and training data might not necessarily have the best incidence of relevant models. If the design objective of the metabolic study is to get much faster models than those in the test and training data, then these special generators could be leveraged. Since, generation is extremely efficient with neural networks, one could create large synthetic datasets in seconds and curate for the models with the desired ‘faster’ dynamics.

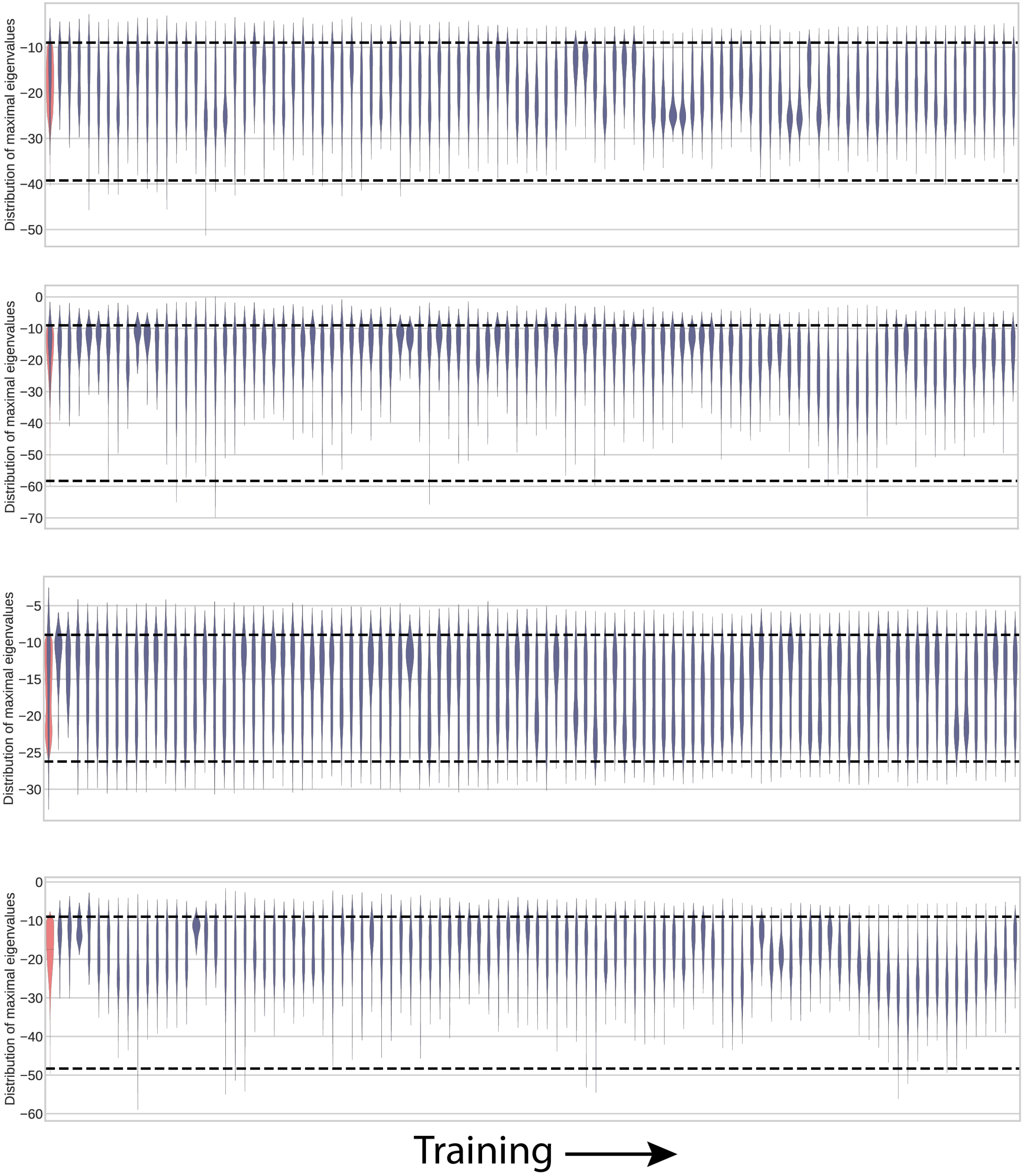

Supplementary Figure 19: Distribution of maximum eigenvalues of relevant generated kinetic models (purple violins) over the course of training (left to right) for a) Physiology 1, b) Physiology 2, c) Physiology 3, d) Physiology 4, compared to the training and test data (orange violin, both distributions overlap). The dashed lines indicated the maximums and minimums of the test and training datasets.

References

1. King, Z. A. *et al.* S1-Escher: A Web Application for Building, Sharing, and Embedding Data-Rich Visualizations of Biological Pathways. *Plos Comput Biol* 11, e1004321 (2015).

2. Kingma, D. P. & Welling, M. Auto-Encoding Variational Bayes. *Arxiv* (2013).

3. Weiss, K., Khoshgoftaar, T. M. & Wang, D. A survey of transfer learning. *J Big Data* 3, 9 (2016).

4. Karras, T. *et al.* Training Generative Adversarial Networks with Limited Data. *arXiv* cs.CV, (2020).
